# A lightweight edge-deployable detection method for sugarcane stem nodes in intelligent seed cane cutting equipment

**DOI:** 10.3389/fpls.2026.1934317

**Published:** 2026-09-30

**Authors:** Zhaojun Niu, Huadong Xiao, Zebin Wu, Bohao Lin, Chang Ge, Lijiao Wei, Zexin Chen, Weihua Huang, Shuo Wang, Dongjie Du, Zhenhui Zheng

**Affiliations:** 1 Agricultural Machinery Research Institute, Chinese Academy of Tropical Agricultural Sciences, Zhanjiang, China; 2 College of Engineering, South China Agricultural University, Guangzhou, China; 3 College of Mathematics and Informatics, South China Agricultural University, Guangzhou, China

**Keywords:** edge deployment, intelligent seed cane cutting, lightweight architecture, motion blur robustness, object detection, sugarcane stem node, YOLOv11

## Abstract

**Introduction:**

Accurate detection of sugarcane stem nodes is essential for intelligent seed cane cutting, yet existing detectors are often limited by large parameter counts, small target feature loss, and performance degradation under motion blur, making them difficult to deploy on edge devices.

**Methods:**

This study developed SAD-YOLOv11, a lightweight and accurate detector based on YOLOv11, incorporating three modifications. These include an average pooling downsampling module named ADown for reducing computational cost while preserving edge features, a self attention based AIFI module for global contextual modeling, and a SEAMHead detection head with joint channel and spatial attention for adaptive feature calibration. The model was evaluated on a custom sugarcane stem node dataset.

**Results:**

The model achieved a precision of 95.92%, a recall of 95.66%, and an mAP50 of 97.57%, maintaining detection accuracy at a level comparable to the baseline YOLOv11 while reducing parameter count and FLOPs by 18.13% and 17.55%. Under physically captured motion blur conditions, the model maintained more stable detection than the baseline, with the advantage becoming more pronounced as blur severity increased. Deployment on the Jetson Orin NX platform with TensorRT-FP16 quantization achieved a throughput of 37.1 FPS with a zero frame drop rate at a 30 FPS input frame rate, and compressed the engine size by 46.7%.

**Discussion:**

These results suggest that SAD-YOLOv11 achieves a sound balance between detection accuracy and computational efficiency suitable for intelligent seed cane cutting equipment.

## 1 Introduction

Sugarcane is a major global crop for sugar production and bioenergy, and it plays an important strategic role in sugar supply and agricultural development (Ma et al., 2014). However, the seed cane cutting process in current sugarcane harvesting operations remains predominantly dependent on manual labor. This approach is labor intensive, inefficient, and associated with safety risks. In addition, conventional machinery typically operates without real-time visual guidance, which frequently damages healthy buds at the stem nodes, directly reducing the survival rate and germination quality of seed cane setts (Alencastre-Miranda et al., 2018). As agricultural labor becomes increasingly scarce and smart agriculture continues to advance, the replacement of manual operations with machine vision and automated equipment, such as intelligent seed cane cutters and sugarcane harvesters, has emerged as a promising direction for modernizing the sugarcane industry. In automated seed cane cutting, rapid and accurate localization of sugarcane stem nodes is a prerequisite for allowing the cutter to avoid cane buds and maintain cutting quality (Nawi et al., 2014). This direction is further supported by the expanding adoption of machine vision and deep learning techniques across various agricultural tasks, including UAV-based crop counting (Zhu et al., 2026), visual phenotypic assessment (Palacios et al., 2022), and image quality enhancement (Siok et al., 2020), which collectively indicate the technical readiness of visual intelligence methods for agricultural automation. Therefore, the development of efficient and lightweight visual detection methods for sugarcane stem nodes holds practical significance for advancing full-process mechanization and intelligent production in sugarcane cultivation.

Early studies on sugarcane stem node recognition relied on traditional image processing techniques, including convolution-based intensity thresholding (Moshashai et al., 2008), vertical projection function extrema (Chen et al., 2020), gradient features (Yang et al., 2020), contour analysis (Pothula et al., 2015), and combinations of mathematical morphology, edge operators, and machine learning classifiers under structured laboratory conditions (Lu et al., 2010; Huang et al., 2013; Zhang et al., 2016). However, these methods depend heavily on manually engineered visual features and exhibit limited generalization and real-time performance in high-throughput conveyor belt scenarios, where interference from belt textures, stalk posture variations, and motion blur substantially degrade feature reliability. Deep learning methods, which learn task-relevant features directly from data, have accordingly attracted growing attention in sugarcane stem node recognition research.

In recent years, deep learning methods based on convolutional neural networks have achieved notable progress in sugarcane stem node detection. Chen and colleagues confirmed the feasibility of combining data augmentation with deep learning for stem node recognition (Chen et al., 2021), and Zhou, Wang, and other researchers further explored deep learning applications in intelligent seed cane cutting systems (Zhou et al., 2020; Wang et al., 2022). Multiple research groups have successively improved YOLO-based models, including YOLOv3, YOLOv5, YOLOv7, YOLOv8, and YOLOv11, through the introduction of attention mechanisms or feature pyramid optimization, yielding improved detection accuracy under diverse conditions (Zhou et al., 2022; Wen et al., 2023; Xie et al., 2023; Zheng et al., 2024; Wen et al., 2025). Similar strategies have been applied to related tasks such as sugarcane stalk base and bud recognition (Zhang et al., 2024; Li H. et al., 2025; Maurya et al., 2025). Despite these advances, deep learning methods optimized for high precision are frequently characterized by large parameter counts and considerable computational costs, making them difficult to deploy on edge devices with constrained computing resources (Yu et al., 2023; Zheng Z. et al., 2025; Zheng ZH. et al., 2025).

When general-purpose large-scale models such as YOLOv11 are applied to sugarcane stem node detection in semi-structured conveyor belt environments, several limitations become apparent. First, intelligent cutting equipment typically relies on resource-constrained embedded edge platforms, such as the NVIDIA Jetson Orin NX and Jetson Nano, for cost-effective deployment. These devices typically deliver computing power in the range of 0.5 to 100 TOPS with correspondingly limited power budgets. Conventional large-scale models with more than 25 million parameters and 80 billion floating-point operations struggle to sustain real-time inference speeds above 30 frames per second, which represents the minimum throughput requirement for high-throughput seed cane cutting pipelines. Second, dynamic motion blur resulting from relative movement between the camera and the targets during high-throughput transportation can degrade image quality by attenuating high-frequency edge features that are critical for localizing small targets such as stem nodes (Gao et al., 2024). This degradation increases the difficulty of accurate target identification and contributes to a noticeable decline in the detection performance of conventional models. Although the robustness experiments in this study were conducted using motion blur images captured through handheld camera acquisition rather than through actual conveyor belt transport, the resulting image degradation shares the common characteristic of attenuating high-frequency edge features, which is the primary mechanism by which motion blur degrades small target detection performance.

In addition to the hardware and blur constraints discussed above, sugarcane stem nodes on conveyor belts present inherent detection challenges. The nodes appear as densely distributed small targets whose fine-grained spatial features can be eliminated by repeated downsampling in general-purpose models. The mesh conveyor belt background introduces repetitive structural patterns that can be confused with stem node features, and the low contrast between node boundaries and adjacent internode surfaces further reduces target salience. These challenges bear similarity to those reported in other crop detection tasks, where task-specific reconstruction of YOLO-based architectures has proven effective in improving both detection accuracy and deployment efficiency. For example, researchers such as Chen, Liu, and Yang developed lightweight architectures for greenhouse peppers, carrot seedlings, and maize root stubble, respectively, and deployed them on edge devices (Chen et al., 2025; Liu et al., 2026; Yang et al., 2026). Similarly, Luo et al (Luo ZP. et al., 2026). proposed LMD-Net by introducing lightweight downsampling, multi-scale feature extraction, and attention-enhanced detection modules into YOLOv13 for rice disease detection under complex field conditions. Additionally, Huang, Luo, and Zhang introduced attention mechanisms and low-light enhancement modules for litchi, citrus, and apple leaf detection, achieving improved precision and robustness under complex operating conditions (Huang MJ. et al., 2025; Luo YM. et al., 2026; Zhang and Wang, 2026). These studies collectively suggest that customized architectural modification of YOLO models, rather than direct application of general-purpose configurations, is a practical strategy for addressing crop-specific detection challenges in resource-constrained environments, which provides methodological reference for the approach adopted in this study.

In light of the above limitations, this study identifies two key requirements that existing methods have not yet simultaneously satisfied (Wei et al., 2025). The first is structural lightweighting compatible with edge platform deployment without sacrificing fine-grained feature representation for small targets. The second is robust feature enhancement that maintains detection accuracy under motion blur and complex background conditions. Addressing both requirements within a unified model architecture remains a research gap. To address these gaps, this study developed SAD-YOLOv11, a customized lightweight and highly accurate detector specifically designed for intelligent seed cane cutting equipment. The main contributions of this paper are explicitly linked to the aforementioned technical limitations and are summarized as follows:

1. A lightweight and accurate object detection model, termed SAD-YOLOv11, is constructed by coordinating three existing modules, each selected to address a specific technical challenge identified in the preceding analysis. An average pooling-based downsampling module, ADown, is integrated at the shallow backbone stages to reduce computational redundancy while preserving fine-grained spatial features of small targets. An attention-based intra-scale feature interaction module, AIFI, is used to replace the original SPPF module in the deep backbone layers to strengthen global contextual modeling and suppress background noise. A spatial-attention-enhanced detection head, SEAMHead, is adopted to adaptively calibrate feature importance for improved target discrimination under motion blur conditions. The contribution of this work lies in the task-oriented selection and coordinated positioning of these modules across different network stages to form a complementary pipeline for sugarcane stem node detection on conveyor belts.
2. Comprehensive experiments were conducted under practical operating conditions. All experiments were repeated five times with different random seeds, with mean and standard deviation reported in the corresponding tables to ensure statistical reliability. A unified interpretation framework is adopted throughout the results sections. The proposed model was evaluated on a custom sugarcane dataset and validated on motion blur test subsets representing mild and severe conditions. The results indicate that SAD-YOLOv11 maintains competitive detection accuracy relative to the baseline YOLOv11 and other mainstream models, while demonstrating improved robustness under severe motion blur conditions.
3. The practical deployability of the model was verified through physical edge deployment and optimization experiments on an NVIDIA Jetson Orin NX platform. Using TensorRT-FP16 quantization, the proposed method achieved real-time inference throughput compatible with the target input frame rates for industrial seed cane cutting equipment while reducing hardware resource consumption, demonstrating the feasibility of edge-integrated deployment under the tested experimental conditions.

## 2 Materials

### 2.1 Image acquisition

The sugarcane image acquisition experiment was conducted on 4 June 2025 at the sugarcane experimental base of the Agricultural Machinery Research Institute, Chinese Academy of Tropical Agricultural Sciences, in Zhanjiang, Guangdong Province, China. The site is located at 110.27° E and 21.17° N, with an elevation of 33 m. The sugarcane samples used in this study belonged to a common commercial variety with light yellow-green rind. Some surfaces carried small amounts of soil residue, which introduced minor visual occlusion consistent with typical post-harvest field conditions. During image acquisition, the sugarcane stalks were placed on a mechanical device comprising a metal frame and a green mesh conveyor belt, as shown in **Figure 1**. Uniform industrial lighting was applied to ensure that the sugarcane rind exhibited sufficient contrast against the green mesh background for manual annotation and subsequent model training.

**Figure 1:**
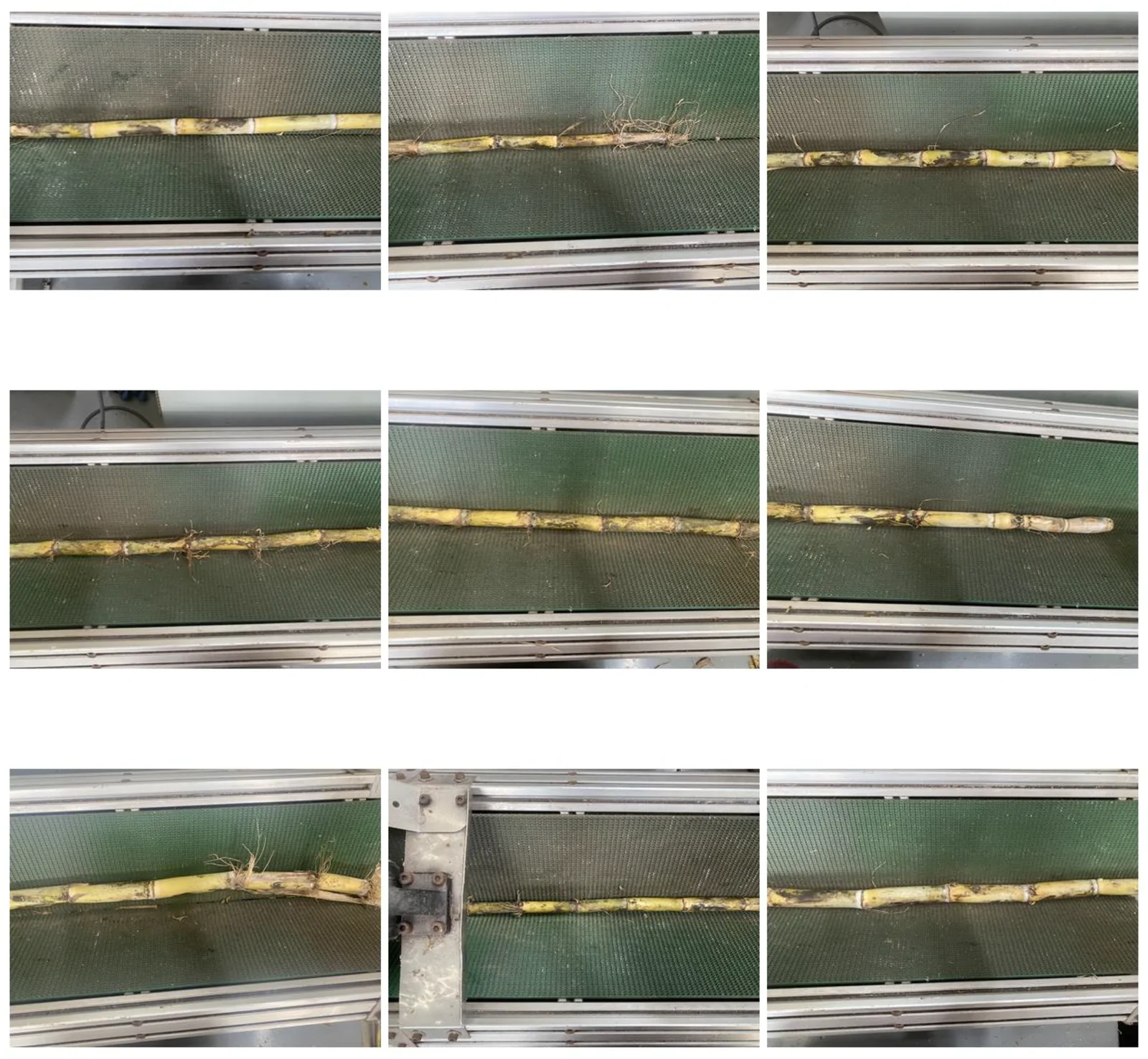
Sugarcane image acquisition experiment.

Images were captured using an iPhone 11 smartphone at a resolution of 4032 × 3024 pixels and stored in JPEG format. The choice of a consumer-grade device rather than an industrial camera was intended to reflect the cost-sensitive deployment conditions of agricultural equipment. A total of 1200 valid images were obtained. Each image contained 3 to 8 stem node targets, yielding approximately 6400 sugarcane stem node instances in total. Given the single-class nature of the detection task and the typical data requirements of YOLO-based architectures, this acquisition yielded a dataset of adequate scale for model training, validation, and testing.

### 2.2 Dataset construction

Dataset quality directly influences both training outcomes and practical deployment performance. A total of 1200 images were captured, each depicting a distinct sugarcane stalk placed on a stationary conveyor belt. During image acquisition, each sugarcane stalk was photographed only once, establishing a one-to-one correspondence between the captured images and the physical stalks. The 1200 images therefore correspond to 1200 independent sugarcane stalks. The dataset was divided at the image level into 960 training images, 120 validation images, and 120 test images using a random partitioning strategy with a ratio of 8:1:1 (Shorten and Khoshgoftaar, 2019; Lu et al., 2022). Since each stalk is represented by exactly one image, image-level partitioning is equivalent to stalk-level partitioning, ensuring that no sugarcane stalk appears in both the training and test subsets.

LabelImg, a widely used annotation tool for object detection, was employed for manual annotation. The generated label files contained the class labels and normalized bounding box coordinates for each sugarcane stem node and were saved in standard YOLO-compatible TXT format (Sun et al., 2022). The detection task involves a single object class, namely sugarcane stem node, which corresponds to the localization requirement of the intelligent seed cane cutting application. A detailed annotation protocol was established prior to labeling to ensure consistency across the dataset. The annotator was instructed to draw the tightest possible axis-aligned rectangular bounding box that fully encloses the visible node region, including the slightly raised transition zone at the upper and lower boundaries where the node meets the adjacent internode, while minimizing the inclusion of surrounding background area.

Candidate targets were excluded from annotation under three conditions. The first was when the visible node area constituted less than approximately 30% of the expected full region, typically due to occlusion by the mesh conveyor belt, overlapping stalks, or accumulated debris. The second was when more than approximately 40% of the node extended beyond the image frame boundary. The third was when the node could not be visually differentiated from the surrounding internode surface despite repeated inspection under magnified viewing conditions. Several visually challenging cases were documented during annotation, including stem nodes exhibiting very low contrast against the internode surface, nodes partially obscured by soil residue or residual fiber fragments, and nodes truncated at the image edges. These cases collectively account for approximately 5% to 8% of the total annotations and reflect the inherent boundary-level difficulty of the detection task.

To evaluate annotation quality, a stratified random subset of 150 images was independently re-annotated by a second annotator with equivalent domain knowledge, who had no access to the first annotator’s labels. Using an IoU threshold of 0.50 as the matching criterion, the inter-annotator agreement rate was 97.3%. The disagreement cases were jointly reviewed and resolved through consensus discussion to produce the final ground truth labels.

To provide quantitative context for the model design requirements described in the Methodology section, a statistical analysis of the annotated targets was conducted from three perspectives. First, regarding target size, over 85% of the sugarcane stem node bounding boxes occupy less than 5% of the total image area, which classifies them as small targets that are highly susceptible to feature loss during downsampling. Second, the aspect ratio, namely the ratio of width to height, of the bounding boxes is densely concentrated between 0.3 and 0.5, representing approximately 1:3 to 1:2. This reflects the narrow and elongated geometric characteristics of stem nodes. Finally, the spatial distribution analysis indicates that the targets are continuously and densely aligned along the horizontal axis of the conveyor belt. These objective statistical characteristics indicate the need for a model capable of retaining fine-grained features of small targets and discriminating between densely distributed objects.

## 3 Methodology

### 3.1 YOLOv11 object detection model

Given the dual requirements of detection accuracy and edge deployment efficiency established in the Introduction, this section presents the model architecture that forms the basis for the proposed improvements. Current object detection methods can be divided into two-stage frameworks represented by the R-CNN family and single-stage frameworks represented by the YOLO family. Two-stage detectors generally achieve higher localization precision through region proposal mechanisms, but they require more computing power and incur longer inference latency, which limits their deployment on edge devices where inference speed must exceed 30 FPS to match production line throughput. By contrast, single-stage frameworks eliminate the region proposal stage and process each image in a single forward pass, offering a more favorable balance among real-time performance, model size, and detection accuracy. Within the single-stage category, the YOLO family has undergone rapid architectural iteration and benefits from a large open-source ecosystem, making it a practical starting point for task-specific adaptation in agricultural vision applications.

Among the available YOLO models at the time of this study, YOLOv11 was selected as the baseline owing to its balanced trade-off between detection performance and computational cost, which aligns with the resource constraints of edge deployment for sugarcane stem node detection (Bai et al., 2023). Compared with YOLOv8, YOLOv11 introduces several architectural refinements. Specifically, it replaces the C2f module with the lighter C3k2 module in the backbone and neck, embeds the pointwise spatial attention mechanism through the C2PSA module, and reconstructs the classification branch in the detection head using depthwise separable convolution. These modifications collectively reduce parameter count while strengthening local feature capture and spatially adaptive feature selection. The following subsections describe these architectural components in detail and provide the foundation for the three targeted modifications introduced in this study.

#### 3.1.1 Backbone and neck networks

In the backbone and neck of YOLOv11, the original C2f module is replaced by the C3k2 module. This change reduces the number of parameters and the computational burden while strengthening the network’s ability to capture local detail. Compared with the PAFPN neck used in YOLOv8, the YOLOv11 neck further improves computational efficiency and feature adaptation, which makes it better suited to lightweight deployment under limited computing resources. YOLOv11 also introduces the C2PSA module into the backbone. Built upon the C2f structure, C2PSA embeds the pointwise spatial attention mechanism PSA. As shown in **Figure 2**, C2PSA first compresses the channels of the input feature map through convolution. A Split module then divides the features evenly into two branches. One branch is retained directly, while the other is passed to a PSA Block consisting of a configurable number of PSA units for attention enhancement. The output of each PSA unit is then concatenated with the retained branch, and a final convolution generates the output features of the module. By incorporating self-attention, C2PSA enables the model to dynamically emphasize target region features while suppressing redundant information in complex backgrounds, thereby compensating for the limited spatially adaptive capability of the purely convolutional structure in YOLOv8.

**Figure 2:**
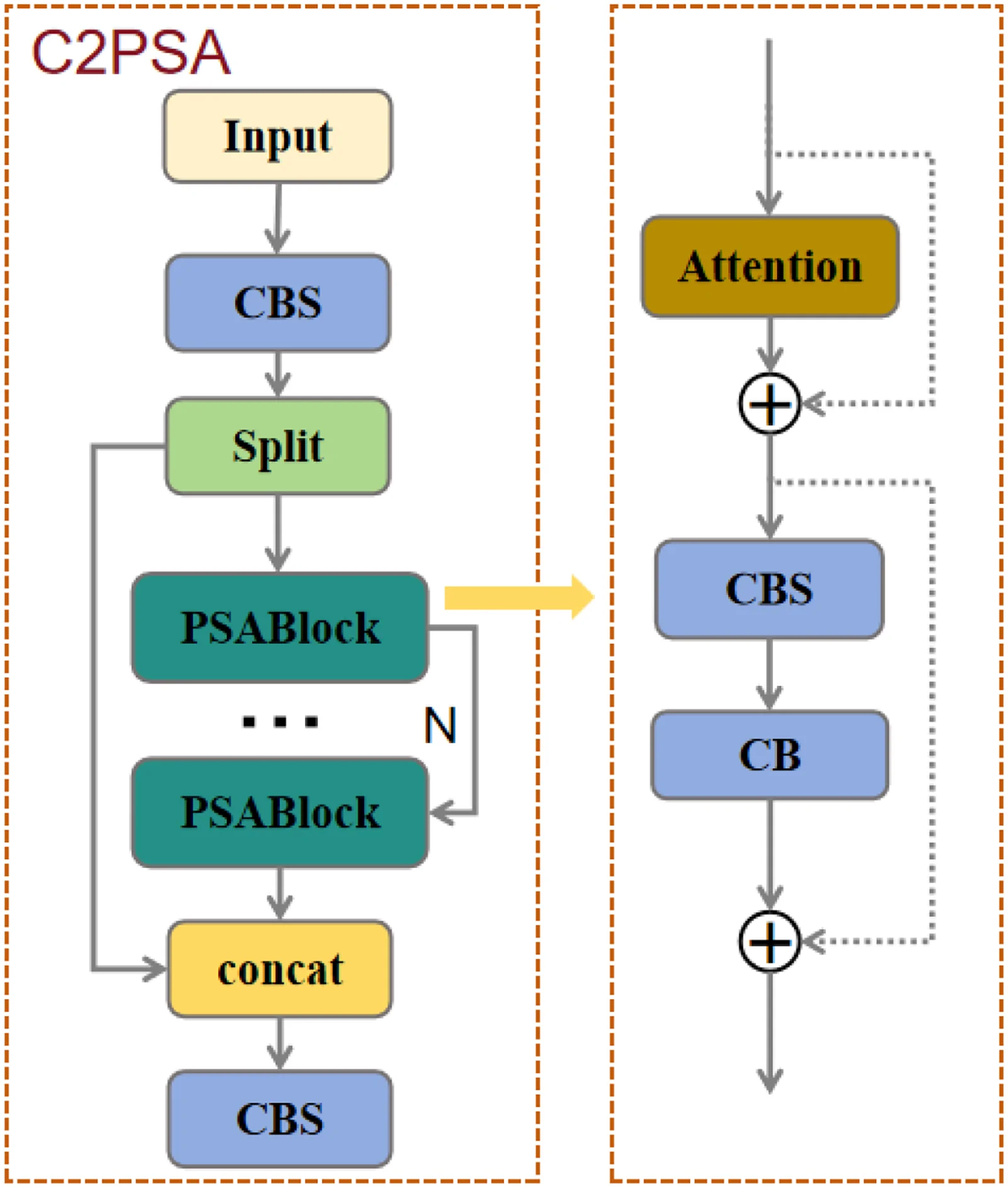
Structure of the C2PSA module.

#### 3.1.2 Prediction network

Object detection algorithms can also be divided into anchor-based and anchor-free methods according to whether they rely on predefined anchor boxes. YOLOv11 continues the anchor-free and decoupled head design used in YOLOv8, and focuses on optimizing the category prediction branch. In particular, it replaces conventional convolution with depthwise separable convolution, thereby reconstructing the classification branch. Compared with the decoupled head in YOLOv8, YOLOv11 reduces redundant computation in the detection head and substantially lowers computational cost. This change also alleviates feature conflict between the classification and regression branches, and better satisfies the computing constraints of edge deployment while preserving bounding box regression accuracy and convergence speed.

### 3.2 Improvements in the SAD-YOLOv11 model

Among the available YOLOv11 size variants, a preliminary evaluation was conducted by training three variants, namely YOLOv11m, YOLOv11l, and YOLOv11x, on the same sugarcane stem node dataset under identical experimental settings. These three variants were selected to span a range from medium to extra-large scale, providing a lighter reference point and a heavier reference point relative to the candidate baseline. The results showed that YOLOv11m achieved a mAP_50_ of 97.42% with 20.03M parameters and 67.6G FLOPs but exhibited relatively unstable performance with the lowest recall of 93.83% and the lowest mAP_50–95_ of 54.14% among the three variants. YOLOv11l achieved a mAP_50_ of 97.50% with 25.28M parameters and 86.6G FLOPs, outperforming YOLOv11m on all four accuracy metrics including a recall advantage of 1.70 percentage points. YOLOv11x achieved a comparable mAP_50_ of 97.41% despite having a parameter count of 56.83M and FLOPs of 194.4G, representing more than twice the computational cost of YOLOv11l without accuracy improvement. Based on these preliminary results, YOLOv11l was selected as the baseline for two considerations. First, the preliminary evaluation demonstrated that YOLOv11m exhibited lower recall and lower mAP_50–95_ than YOLOv11l, suggesting that the medium-scale variant may not provide sufficient representational stability for this detection task. YOLOv11l achieved the highest mAP_50_ among the three evaluated variants and outperformed YOLOv11m on all four accuracy metrics. Second, YOLOv11l with 25.28M parameters and 86.6G FLOPs represents a scale from which coordinated architectural modifications can demonstrate meaningful absolute compression while retaining the channel dimensions needed for the self-attention operations in the AIFI module and the MLP-based channel dependency learning in the SEAMHead module. The resulting SAD-YOLOv11 achieves 20.70M parameters and 71.4G FLOPs, which is comparable in parameter count to YOLOv11m at 20.03M. In the preliminary evaluation, YOLOv11m achieved a recall of 93.83%, whereas SAD-YOLOv11 achieved a five-run mean recall of 95.66%. Although these values are derived from different evaluation protocols, the magnitude of the difference suggests that the coordinated module design provides a more effective approach than simply using a medium-scale model, as the accuracy gains from the proposed modules compensate for the slight increase in parameter count relative to YOLOv11m. To ensure a fair and consistent comparison, all comparison models in Section 5.3 were subsequently evaluated using their corresponding large-scale variants. The selected baseline nevertheless exhibited a relatively large parameter count and high computational cost, and the specific limitations of computational redundancy, limited global context modeling, and insufficient feature discrimination under motion blur identified in the Introduction still remained.

To address these limitations, three existing modules were selected and integrated in a coordinated manner across different network stages, following a pipeline of lightweight feature extraction, semantic enhancement, and focused feature discrimination. The selection of each module was guided by the specific requirements of sugarcane stem node detection on conveyor belts, and the positioning of each module was determined by its functional role within the pipeline. At the shallow feature extraction stage, the ADown module reduces spatial dimensions and parameter redundancy while preserving edge features of small targets. This stage necessarily reduces the local receptive field, which limits the model’s ability to relate target features to their broader spatial context. The AIFI module is subsequently deployed at the deepest layer of the backbone to establish global semantic dependencies that span beyond the local receptive field constrained by prior downsampling operations. At the prediction stage, SEAMHead applies channel-spatial joint attention to the fused feature maps, selectively strengthening target-relevant features and suppressing residual background interference before bounding box regression. The resulting pipeline is designed to address the three specific challenges identified in the Introduction, namely computational redundancy, limited global context modeling, and insufficient feature discrimination under motion blur. The overall effect is improved feature discrimination for sugarcane stem nodes without introducing feature conflicts or excessive computational overhead. The improved model balances the real-time demands of edge deployment with the need for high accuracy, and provides technical support for intelligent visual systems for sugarcane stem nodes. The improved network architecture is shown in **Figure 3**.

**Figure 3:**
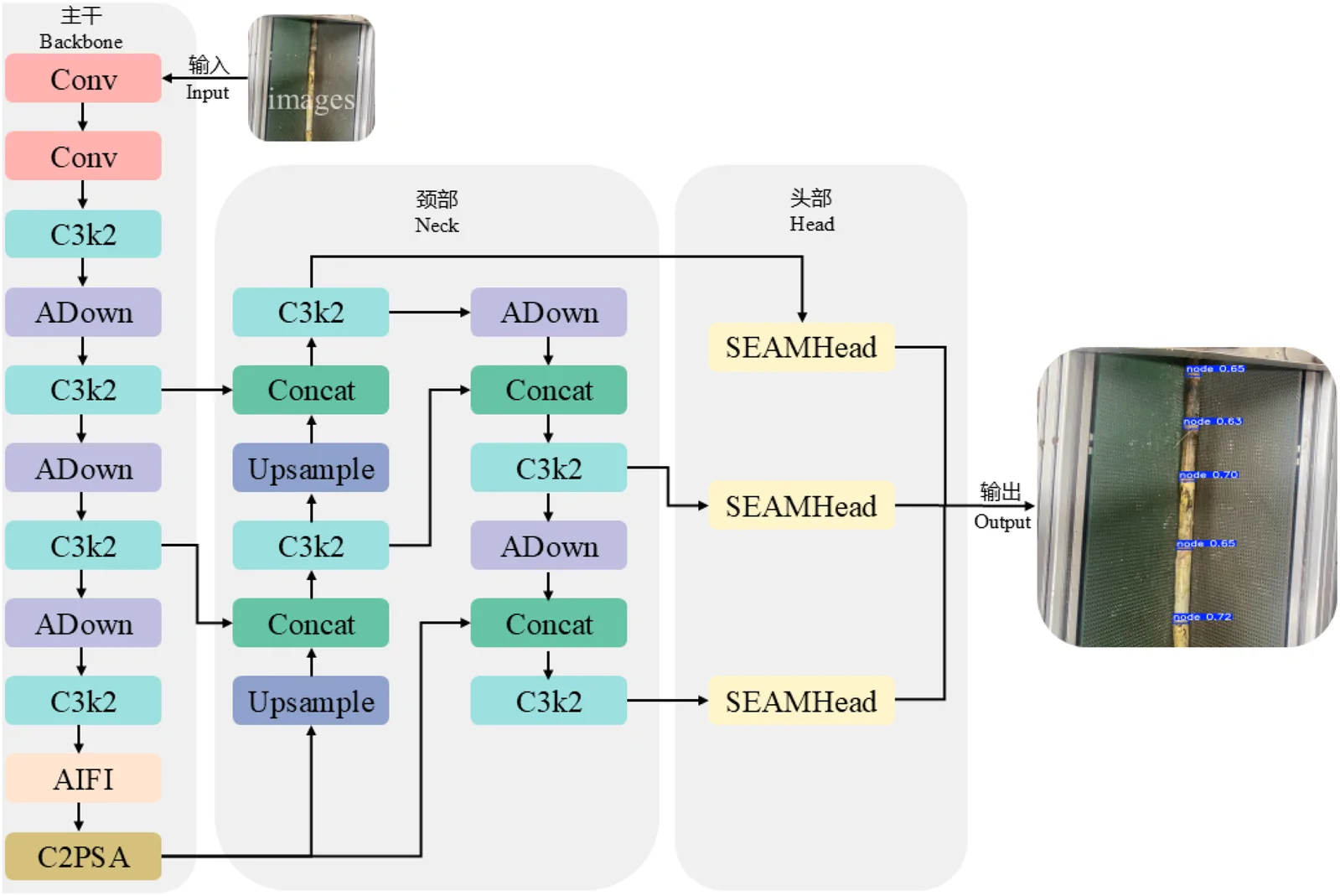
Network architecture of the improved SAD-YOLOv11 model.

#### 3.2.1 ADown average pooling downsampling module

The original convolutional downsampling in YOLOv11 reduces the size of feature maps, but it can also cause loss of fine detail, which is unfavorable for small target detection. Its computational burden also constrains deployment efficiency on edge devices. To address this issue, the ADown module was adopted to replace the original downsampling layers. As shown in **Figure 4**, ADown first uses average pooling to extract smooth global background information from the feature map, which reduces the influence of noise and abnormal pixels during downsampling. It then adopts a parallel two-branch structure to lower computational complexity while retaining as much useful information as possible (Wang et al., 2024). In the first branch, a 3 × 3 convolution is used to extract deeper features. In the second branch, max pooling captures local extreme responses to strengthen salient information such as edges and corners. A subsequent 1×1 convolution performs channel transformation to preserve key discriminative cues (Zhang and Jiang, 2025). The outputs of the two branches are finally concatenated along the channel dimension to combine global contextual information with local salient responses (Gu et al., 2025).

**Figure 4:**
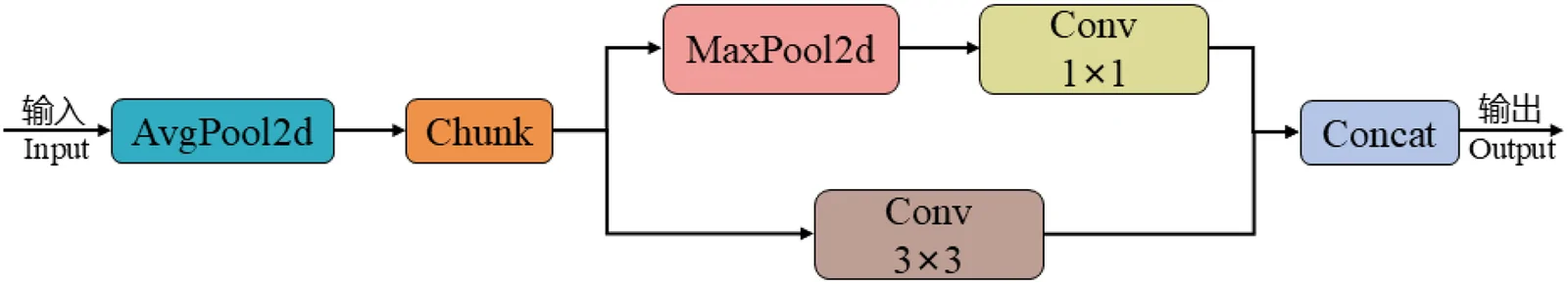
Structure of the ADown module.

By using pooling operations to replace the stride-2 convolution employed in the original YOLOv11 downsampling layers, ADown reduces floating-point operations and parameter count while the two-branch structure maintains complementary feature representations at each spatial reduction step (Sun et al., 2025). At the same time, the complementary two-branch mechanism helps alleviate feature attenuation during downsampling and supports efficient lightweight compression (Ren et al., 2025). Overall, ADown reduces parameter count and FLOPs at the shallow backbone stage.

#### 3.2.2 AIFI module for attention based intra scale feature interaction

In YOLOv11, the backbone employs the SPPF module to aggregate features through parallel multiscale max pooling, which expands the receptive field and enhances multiscale feature representation. However, SPPF relies on local max pooling operations with fixed kernel sizes, which means its receptive field expansion is bounded by the cumulative pool size rather than by the spatial extent of the feature map. For sugarcane stem node detection, this bounded receptive field makes it difficult to capture contextual relationships between a stem node and distant conveyor belt regions that share similar repetitive textures, which is relevant to distinguishing true targets from background noise (Zhao et al., 2024). In addition, its multi-branch pooling structure introduces redundant computation when deep feature maps are processed, which reduces deployment efficiency on edge devices.

To address this issue, AIFI, an attention-based intra-scale feature interaction module, was adopted to replace the SPPF module. As shown in **Figure 5**, AIFI uses self-attention to perform feature interaction within the same scale, with emphasis on global fusion and information exchange among features at that scale. This design helps build a global receptive field and suppress interference from complex backgrounds (Liu et al., 2024). During forward propagation, AIFI first receives the deep feature map from the backbone, flattens it to match the Transformer input format, and retains local spatial information through positional encoding. The feature vectors are then passed through a multi-head self-attention structure in the Transformer encoder, where attention weights across all positions are computed to model global information and enable feature interaction (Ling et al., 2024). The output features then pass through residual connections, layer normalization, and a feed-forward network for nonlinear transformation and feature enhancement, followed by another round of residual connection and layer normalization. The features are finally reshaped into a two-dimensional tensor for output (Zhang H. et al., 2025).

**Figure 5:**
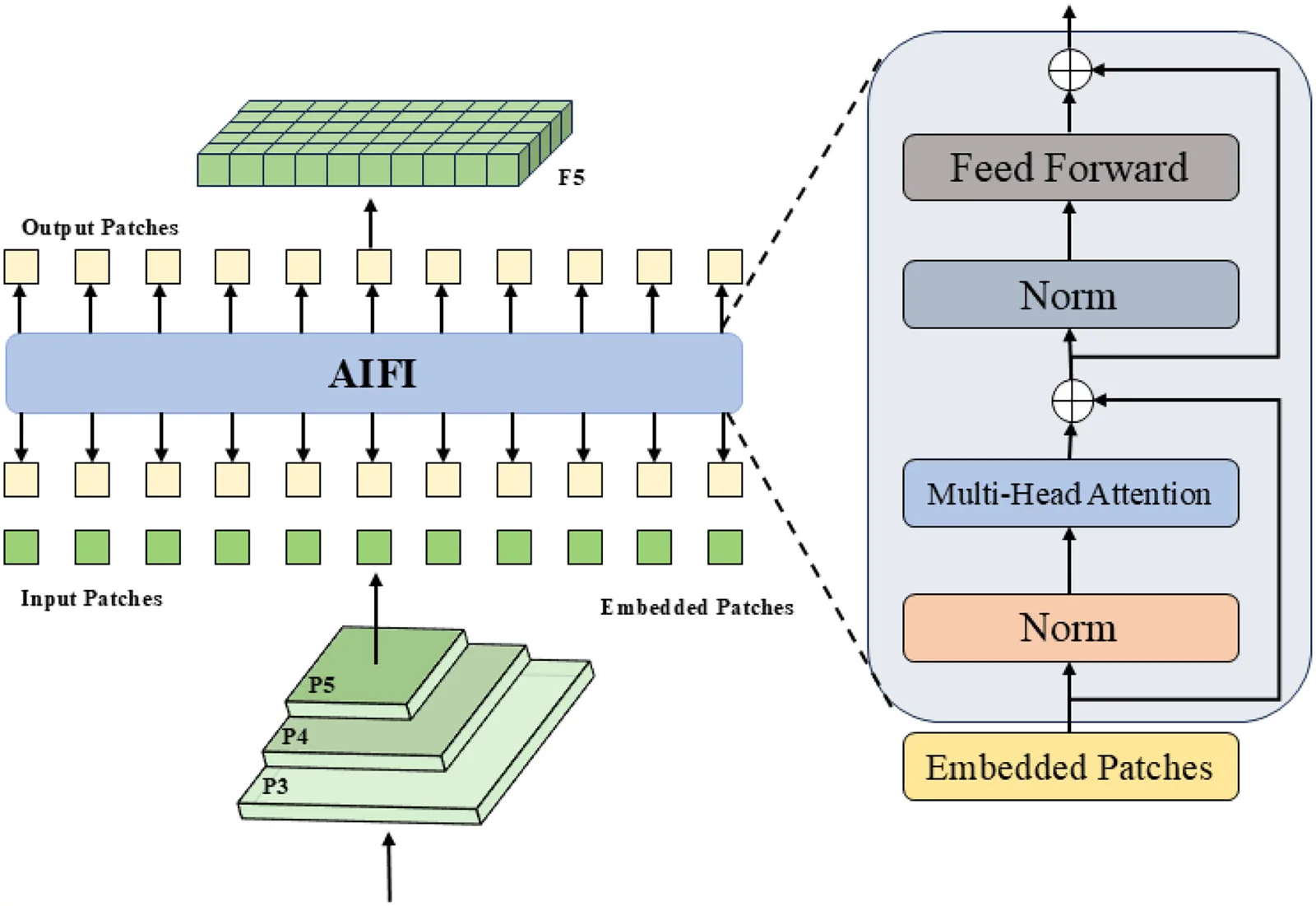
Structure of the AIFI module.

Agricultural vision tasks frequently involve strong background interference and substantial variation in target scale. At the same time, practical deployment imposes strict computational cost constraints on edge devices. The model therefore needs to balance noise suppression, enhancement of small target features, and computational efficiency. The local pooling operation in SPPF is not well suited to handling background interference. By contrast, the global contextual modeling in AIFI addresses this limitation while remaining compatible with lightweight deployment (Zhang Y. et al., 2025). Accordingly, the SPPF module at the end of the YOLOv11 backbone was replaced with AIFI in this study. The global contextual modeling ability of AIFI in the deep network improves the integrity and discriminability of feature representation. AIFI was positioned at the deepest backbone layer following the ADown modules, where its self-attention mechanism operates on the compressed feature maps produced by the preceding stages.

#### 3.2.3 SEAMHead detection head with channel-spatial joint attention enhancement

The Detect head in YOLOv11 reconstructs the category prediction branch using depthwise separable convolution, achieving a trade-off between bounding box regression accuracy and model lightweighting. However, it still relies primarily on stacked convolutions for feature extraction and lacks an explicit mechanism for evaluating feature importance, which limits performance when targets are degraded by motion blur or occluded by complex conveyor belt backgrounds. To address this limitation, SEAMHead incorporates a channel-spatial joint attention mechanism that performs feature calibration in two stages. The first stage applies depthwise separable convolutions to encode spatial relationships within each feature channel independently, which preserves fine-grained positional information. The second stage applies channel-wise attention to selectively emphasize feature channels that are most relevant to stem node discrimination. This two-stage design is motivated by the physical characteristics of sugarcane stem nodes, whose elongated morphology with aspect ratios concentrated between 0.3 and 0.5 is expected to benefit from spatial positional encoding along the major axis and channel-wise calibration along the minor axis.

To address this issue, the SEAMHead detection head was constructed by incorporating the Scale Enhanced Attention Mechanism. Conventional channel attention modules, exemplified by the SE block, employ a Sigmoid function to constrain channel weights to the range of zero to one, which primarily enables suppression of unimportant channels. SEAM retains the Sigmoid function but additionally applies an exponential mapping to transform the constrained weights into the one to e range, enabling amplification of important target features in addition to suppression of background channels (Fu et al., 2024; Zhou and Jiang, 2025).

As shown in **Figure 6**, the SEAM module decodes features through a defined computational process. First, the input feature map $X\in {\text{R}}^{C\times H\times W}$ undergoes a spatial encoding phase using N residual blocks equipped with depthwise separable convolutions, hereafter referred to as DConv. This step extracts spatial features within each channel independently while maintaining computational efficiency, as expressed in Equation 1 (Yu et al., 2024):

**Figure 6:**
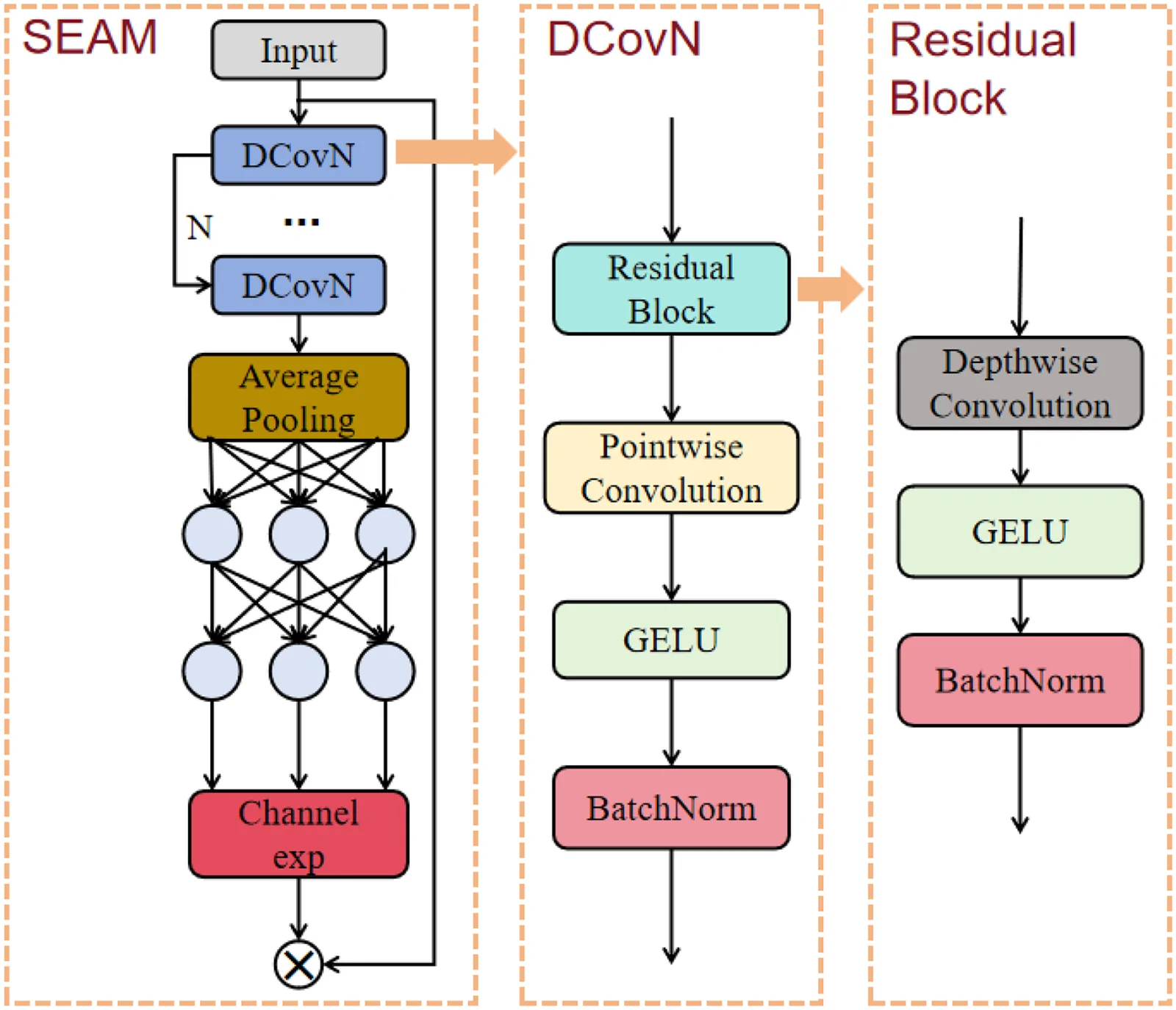
Structure of the improved SEAMHead module.

(1) $$F_{spatial}=\text{DConv}\left(X\right)+X$$

Subsequently, to fuse cross-channel information and strengthen nonlinear relationships, a point-wise convolution (1×1 Conv) is applied. A Global Average Pooling operation then aggregates the spatial description of each channel into a channel descriptor S as shown in Equation 2 $S\in {\text{R}}^{C\times 1\times 1}$:

(2) $$S=\text{GAP}\left({\text{Conv}}_{1\times 1}\left(F_{spatial}\right)\right)$$

The core of the adaptive feature calibration lies in the subsequent weight generation. The descriptor *S* is passed through a multilayer perceptron with two fully connected layers to learn the inter-channel dependencies. An exponential mapping is then applied to generate the final channel weights as expressed in Equation 3:

(3) $$W_{c}=\exp\left(\text{MLP}\left(S\right)\right)$$

This exponential normalization establishes a monotonic mapping that expands the weight value range from (0, 1) to [1, e]. Finally, the optimized channel weights are multiplied element-by-element (⊗) with the spatially encoded features to produce the calibrated output *Y* as shown in Equation 4:

(4) $$Y=W_{c}⊗F_{spatial}$$

The complete computational process of the SEAM module is summarized in Equations 1–4. Through this exponential calibration, SEAM amplifies weak but important target features where the channel weights exceed one, rather than merely suppressing background noise. With SEAMHead integrated into the detection head (Huang JJ. et al., 2025), the model incorporates both spatial feature encoding and channel-wise attention calibration in the prediction stage. This design is intended to improve inference robustness against motion blur without incurring a substantial increase in computational cost (Li Z. et al., 2025; Pan et al., 2025; Wang et al., 2025).

## 4 Experimental environment and evaluation metrics

### 4.1 Platform configuration and parameter settings

To ensure a fair evaluation, all comparative models, including the proposed SAD-YOLOv11 and the baseline models, were trained and evaluated under identical hardware, software, and hyperparameter configurations. The main hardware configuration was as follows. The CPU was an Intel Xeon Gold 6226R running at 3.10 GHz, with 32 physical cores and 64 threads. System memory was 512 GB. The GPU was an NVIDIA GeForce RTX A6000 with 48 GB of memory. The operating system was Ubuntu 20.04.6 LTS with an NVIDIA driver version 580.82.07. Model training was conducted within a conda virtual environment running Python 3.10, using PyTorch 2.10.0 compiled with its bundled CUDA 12.6 runtime and cuDNN 9.1.02. The system-level CUDA Toolkit version was 13.0.88, which is forward-compatible with the PyTorch-bundled CUDA 12.6 runtime through the NVIDIA driver. Additional libraries included OpenCV 4.13.0. All models were implemented using the Ultralytics framework, version 8.4.24, including the baseline YOLOv11l and the comparison models YOLOv5l, YOLOv8l, YOLOv10l, YOLOv12l, YOLOv13l, and YOLOv26l.All models were trained from scratch without loading COCO pretrained weights. The architectural modifications introduced in SAD-YOLOv11, where ADown replaces the original downsampling layers, AIFI replaces the SPPF module, and SEAMHead replaces the original detection head, involve structural changes in layer dimensions that are incompatible with direct weight transfer from standard YOLOv11 checkpoints. The same random initialization approach was applied to all models to ensure a fair comparison. The input image size was uniformly set to 640 × 640 pixels. The maximum number of epochs was set to 10000, and patience in the early stopping mechanism was set to 50. In addition, the batch size was 64, momentum was 0.937, weight decay was 0.0005, and the initial learning rate was 0.01. The learning rate schedule consisted of a linear warmup phase over the first 3 epochs with a warmup momentum of 0.8 and a warmup bias learning rate of 0.1, followed by linear decay to a final learning rate factor of 0.01. Training was conducted using the default data augmentation settings provided by the Ultralytics framework, including mosaic augmentation with a probability of 1.0 throughout the entire training process, horizontal left-right flipping with a probability of 0.5, HSV color augmentation with perturbation ranges of 0.015 for hue, 0.70 for saturation, and 0.40 for value, random translation with a magnitude of 0.1, random scaling with a factor of 0.5, random erasing with a probability of 0.4, and the RandAugment automatic augmentation policy. Vertical flipping, rotation, shear, perspective distortion, mixup, and copy-paste augmentation were disabled. All models were trained under identical augmentation settings to ensure a fair and consistent comparison. The loss function weights were set to 7.5 for bounding box regression, 0.5 for classification, and 1.5 for distribution focal loss. The optimizer was SGD with an initial learning rate of 0.01 and a final learning rate factor of 0.01. A linear warmup was applied over the first 3 epochs with a warmup momentum of 0.8 and a warmup bias learning rate of 0.1, followed by linear decay. Mixed precision training was enabled. Each model was trained five times using different random seeds numbered 0, 42, 123, 999, and 2024 to generate the statistical results reported in **Tables 1**, **2**. The configuration described above was identical across all five runs, with the random seed being the only variable that differed between runs. For the representative training run with random seed 0, the best model checkpoint was selected based on the highest mAP_50_ on the validation set, which occurred at epoch 165 for the baseline YOLOv11 and epoch 150 for SAD-YOLOv11. Training terminated at epoch 184 and epoch 186 for the two models, respectively, due to the early stopping mechanism with a patience of 50 epochs. Across all five runs, the best checkpoint epoch showed limited variation for both models, and the overall convergence behavior was consistent regardless of the random seed. The corresponding mean and standard deviation of the evaluation metrics across all five runs are reported in **Tables 1**, **2**.

**Table 1:** Results of the ablation study.

Model	ADown	AIFI	SEAMHead	Precision/%	Recall/%	mAP_50_/%	mAP_50-95_/%	Parameters/M	FLOPs/G	FPS
1				95.21 ± 0.48	95.53 ± 0.70	97.12 ± 0.52	54.68 ± 1.02	25.28	86.6	154.19 ± 4.33
2	✓			95.83 ± 0.30	95.33 ± 0.51	97.35 ± 0.47	55.01 ± 0.39	19.31	71.6	148.75 ± 2.74
3		✓		96.60 ± 1.13	96.17 ± 0.47	97.87 ± 0.77	55.45 ± 0.82	26.72	86.9	155.92 ± 1.02
4			✓	96.02 ± 0.52	95.89 ± 1.25	98.04 ± 0.57	54.59 ± 1.12	25.21	86.1	155.96 ± 2.59
5	✓	✓		96.11 ± 0.76	95.45 ± 0.77	97.53 ± 0.37	55.02 ± 0.91	20.76	71.9	148.21 ± 1.55
6		✓	✓	95.36 ± 1.28	95.53 ± 0.62	97.36 ± 0.53	54.55 ± 0.51	26.66	86.4	152.56 ± 2.20
7	✓		✓	95.67 ± 0.84	95.95 ± 0.49	97.26 ± 0.45	54.73 ± 0.59	19.24	71.1	141.80 ± 3.39
8	✓	✓	✓	95.92 ± 0.95	95.66 ± 0.89	97.57 ± 0.57	54.93 ± 0.76	20.70	71.4	145.46 ± 2.57

‘√’ indicates that the module was used.

**Table 2:** Performance comparison of mainstream object detection models on the test set.

Model	Precision/%	Recall/%	mAP_50_/%	mAP_50-95_/%	Parameters/M	FLOPs/G	FPS
YOLOv5	96.40 ± 0.39	95.53 ± 1.00	97.86 ± 0.35	54.54 ± 0.74	47.91	114.2	167.73 ± 1.53
YOLOv8	95.79 ± 0.97	95.73 ± 0.37	97.51 ± 0.37	54.94 ± 0.79	39.43	145.2	148.65 ± 3.68
YOLOv10	95.04 ± 1.95	92.98 ± 3.29	96.69 ± 1.68	53.48 ± 2.41	24.31	120.0	142.80 ± 2.07
YOLOv11	95.21 ± 0.48	95.53 ± 0.70	97.12 ± 0.52	54.68 ± 1.02	25.28	86.6	154.19 ± 4.33
YOLOv12	96.10 ± 0.73	95.75 ± 0.54	97.66 ± 0.33	54.09 ± 0.53	25.76	80.8	87.80 ± 2.24
YOLOv13	96.14 ± 0.77	95.70 ± 0.81	97.79 ± 0.51	54.10 ± 0.62	26.88	84.3	67.52 ± 1.26
YOLOv26	95.24 ± 0.51	95.44 ± 0.65	97.15 ± 0.54	54.74 ± 1.09	24.43	86.6	156.75 ± 2.98
SAD-YOLOv11	95.92 ± 0.95	95.66 ± 0.89	97.57 ± 0.57	54.93 ± 0.76	20.70	71.4	145.46 ± 2.57

### 4.2 Evaluation metrics

Model performance was evaluated from two perspectives that correspond to the dual requirements outlined in the Introduction, namely detection accuracy for reliable stem node localization and computational efficiency for edge platform deployment. Detection accuracy was assessed using precision, recall, and mean average precision, which is commonly computed at different Intersection over Union thresholds in object detection. This study reports two variants of mean average precision (Everingham et al., 2009). The first is mAP_50_, which adopts an IoU threshold of 50% and serves as a widely used indicator of overall detection performance under a moderate localization stringency. The second is mAP_50-95_, which computes the average mAP across IoU thresholds from 50% to 95% in increments of 5%. Compared with mAP_50_, mAP_50–95_ imposes stricter requirements on bounding box alignment and is therefore more sensitive to localization errors, providing additional information about the fine-grained positioning capability of the model across a range of localization stringency levels. Computational efficiency was assessed using three complementary metrics. Parameter count reflects the model size and determines storage and memory requirements. Floating-point operations quantify the theoretical computational cost per inference. Inference throughput, measured in frames per second under the hardware configuration described above, reflects the actual runtime speed and is directly relevant to whether the model can meet the throughput demands of high-speed conveyor belt operations. The equations for precision, recall, AP and mAP are standard and are given in Equations 5–8:

(5) $$\text{Precision}=\frac{\text{TP}}{\text{TP}+\text{FP}}$$

(6) $$\text{Recall}=\frac{\text{TP}}{\text{TP}+\text{FN}}$$

(7) $$\text{AP}=\int_{0}^{1}P\left(R\right)dR$$

(8) $$\text{mAP}=\frac{1}{n}\;\sum_{\text{i}=1}^{n}AP_{i}$$

Here, TP denotes the number of true positive detections, meaning predicted boxes that match ground truth boxes with an IoU value at or above the designated threshold. FP denotes the number of false positive detections, meaning predicted boxes that fail to match any ground truth box at or above the designated IoU threshold. This encompasses both predicted boxes whose best IoU with any ground truth box falls below the threshold and predicted boxes generated in images where no corresponding ground truth target exists. FN denotes the number of false negative detections, meaning ground truth sugarcane stem node targets that the model failed to detect. AP denotes the area under the precision-recall curve and reflects the overall detection capability of the model in terms of the trade-off between precision and recall. It should be noted that the precision, recall, and mAP values reported in this study were computed using the default IoU matching criterion implemented in the Ultralytics evaluation framework, which follows the standard COCO evaluation protocol. Under this protocol, predictions are matched to ground truth boxes through a greedy IoU-based assignment at each threshold level, with each ground truth box matched to at most one prediction, and unmatched predictions are classified as false positives.

## 5 Results and analysis

### 5.1 Model training convergence analysis

As shown in **Figure 7**, the convergence curves of SAD-YOLOv11 on the sugarcane dataset are presented for the representative training run with random seed 0. The performance improved steadily as training progressed. During training, the bounding box loss, classification loss, and distribution focal loss for both the training set and the validation set followed a similar pattern, with a rapid initial decrease and a gradual stabilization thereafter. This pattern suggests that the model converged well in both localization and classification, while no obvious overfitting was observed. In terms of evaluation metrics, precision and recall rose rapidly in the early stage and stabilized around 0.90, then exceeded 0.95 later in training. The mAP_50_ increased from about 0.80 at the beginning to above 0.90, while the more demanding mAP_50–95_ metric increased steadily from about 0.40 to above 0.50. Taken together, these results suggest that SAD-YOLOv11 maintained relatively high detection accuracy across different IoU thresholds. The convergence curves therefore indicate that the improved model achieved stable convergence behavior in both localization and classification on the sugarcane detection task, and that no obvious divergence or instability was observed across the training process.

**Figure 7:**
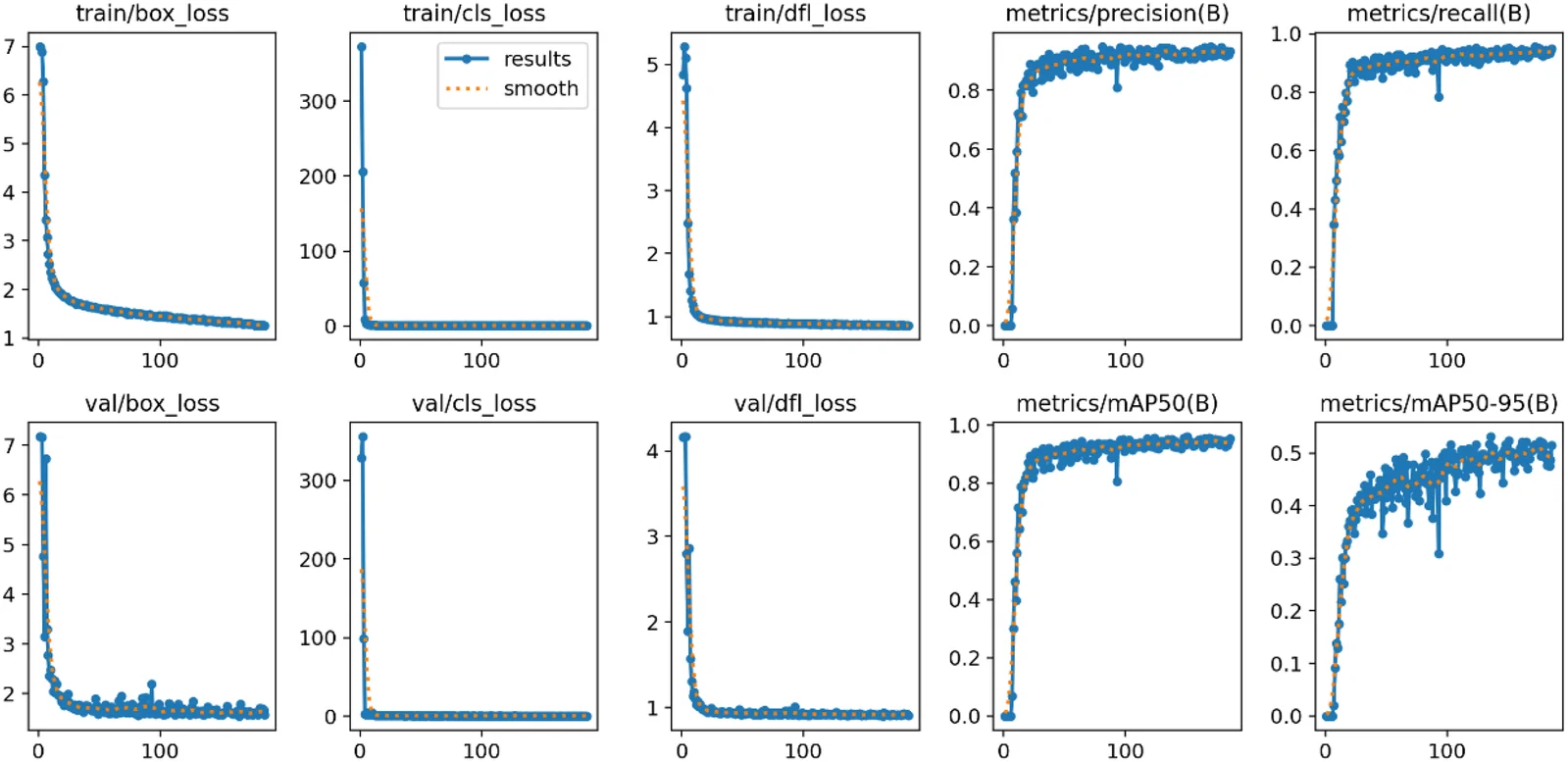
Convergence curves of the sugarcane stem node recognition network for the representative training run with random seed 0.

### 5.2 Ablation study results and analysis

To ensure consistent and transparent interpretation of numerical differences throughout all results sections, the following descriptive judgment framework is adopted. All main experiments in Sections 5.2 and 5.3 are based on five independent training runs with different random seeds numbered 0, 42, 123, 999, and 2024. Mean and standard deviation values for all accuracy metrics are reported in **Tables 1**, **2**. In the following text, mean values are presented for clarity, and readers are referred to the corresponding tables for complete statistical information. Accuracy differences are classified into three categories based on the observed standard deviations reported in **Tables 1**, **2**, where the mean standard deviation across all models for mAP_50_ is approximately 0.5 percentage points. Differences exceeding 1.0 percentage point are reported as meaningful improvements. Differences between 0.5 and 1.0 percentage points are reported as modest improvements. Differences below 0.5 percentage points are reported as comparable performance, as they may fall within the range of normal training variance. This framework is intended to facilitate consistent interpretation of numerical differences and does not constitute a formal statistical significance test. Readers may refer to the mean and standard deviation values in the corresponding tables to assess whether specific differences are likely to exceed normal training variance. This framework is applied uniformly across all results sections. It should be noted that the primary purpose of the ablation study is not to identify modules that individually maximize accuracy, but rather to evaluate whether coordinated lightweight modifications can reduce computational cost without introducing meaningful accuracy degradation. Accordingly, the analysis below emphasizes the trade-off between accuracy preservation and computational cost reduction across all eight variants.

To evaluate the contributions of ADown, AIFI, and SEAMHead to lightweight design and detection accuracy in the sugarcane stem node task, an ablation study was carried out with YOLOv11l as the baseline model. Under identical experimental settings and hyperparameter configurations, the comparison considered both detection accuracy and model complexity. Detection accuracy was evaluated using precision, recall, mAP_50_, and mAP_50-95_, while computational efficiency was evaluated using parameter count, floating-point operations, and inference throughput measured in frames per second. The results are shown in **Table 1** and visualized in **Figure 8**, which presents the detection accuracy, computational cost, inference speed, and module combination matrix across all eight ablation variants. For ease of visualization, **Figure 8** presents the detection accuracy, computational cost, inference speed, and module combination matrix plotted from the single run with random seed 0. Readers are referred to **Table 1** for the complete five-run statistics.

**Figure 8:**
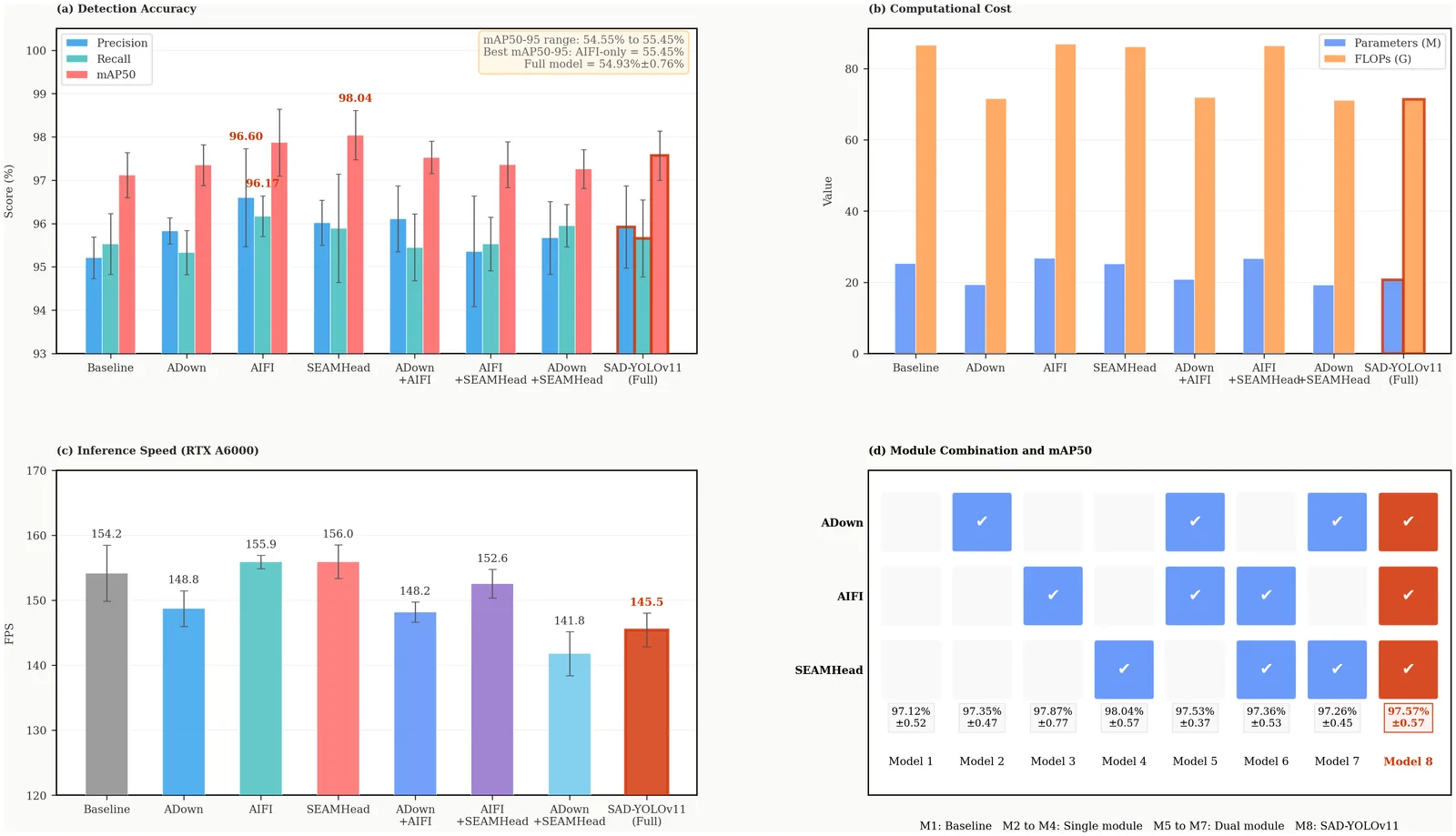
Visualization of the ablation study results across all eight module combinations. All numerical values are averaged over five independent training runs with different random seeds; error bars indicate standard deviation. **(a)** Detection accuracy comparison, showing Precision, Recall, and mAP50 for each variant. **(b)** Computational cost comparison, presenting Parameters and FLOPs for each variant. **(c)** Inference speed comparison on an NVIDIA RTX A6000 GPU, confirming that all variants exceed the 30 FPS real-time threshold. **(d)** Module combination and mAP50 matrix. All variants and metric values correspond directly to those reported in **Table 1**.

According to **Table 1**, compared with YOLOv11, Model 2 with ADown alone reduced the parameter count and FLOPs by 23.59% and 17.32%, respectively. Precision increased by 0.62 percentage points, classified as modest improvement under the unified interpretation framework. Recall decreased by 0.20 percentage points, mAP_50_ increased by 0.23 percentage points, and mAP_50–95_ increased by 0.33 percentage points, all falling within the comparable range. These results confirm that ADown achieves substantial model compression with negligible impact on core detection accuracy. The lightweight foundation established by ADown provides the basis for subsequent module combinations.

Model 3 with AIFI alone introduced an increase in parameter count of 5.72% and an increase in FLOPs of 0.35% relative to the baseline. Precision increased by 1.39 percentage points, exceeding the 1.0 percentage point threshold for meaningful improvement. Recall increased by 0.64 percentage points and mAP_50_ increased by 0.75 percentage points, both classified as modest improvement. mAP_50–95_ increased by 0.77 percentage points, also within the modest improvement range. The inference speed remained essentially unchanged at 155.92 FPS compared with 154.19 FPS for the baseline, confirming that the computational overhead of AIFI is efficiently absorbed within the inference pipeline. These results suggest that the integration of AIFI is associated with improved detection performance while maintaining computational efficiency. The multi-head self-attention mechanism in AIFI operates across the full spatial extent of the feature map, which may provide broader contextual modeling than the local pooling operations in the baseline SPPF module.

With SEAMHead alone, Model 4 showed accuracy differences relative to the baseline that fall within the comparable to modest range under the unified framework. Precision increased by 0.81 percentage points and mAP_50_ increased by 0.92 percentage points, both classified as modest improvement. Recall increased by 0.36 percentage points, within the comparable range. mAP_50–95_ decreased by 0.09 percentage points, also within the comparable range. Parameter count and FLOPs slightly decreased by 0.28% and 0.58%, respectively. These results indicate that the channel-spatial joint attention mechanism in SEAMHead enhances core target features through the Scale Enhanced Attention Mechanism while contributing marginally to model compression. The modest improvement in mAP_50_ is relevant to the seed cane cutting application, as it suggests that SEAMHead contributes to more reliable stem node localization.

When two modules were combined, the interaction effects became more nuanced. In Model 5, which combined ADown and AIFI, precision increased by 0.90 percentage points, classified as modest improvement. Recall decreased by 0.08 percentage points, mAP_50_ increased by 0.41 percentage points, and mAP_50–95_ increased by 0.34 percentage points, all within the comparable range. Model 5 achieved parameter count and FLOPs reductions of 17.88% and 16.97%, respectively, while maintaining mAP_50_ at 97.53%, comparable to the baseline at 97.12%. These results indicate that the combination of ADown and AIFI maintains comparable detection accuracy to the baseline while achieving substantial parameter and FLOPs reductions, consistent with the observation that each module individually preserved or improved accuracy when applied alone.

Model 6 combined AIFI and SEAMHead, introducing a 5.46% increase in parameter count relative to the baseline. Precision increased by 0.15 percentage points, recall remained essentially unchanged at 95.53%, mAP_50_ increased by 0.24 percentage points, and mAP_50–95_ decreased by 0.13 percentage points, all falling within the comparable range. These results suggest that the combination of global contextual modeling from AIFI and channel-spatial attention from SEAMHead yields accuracy comparable to the baseline while introducing a modest increase in parameter count. Importantly, the net parameter count falls below the baseline when the lightweight ADown module is incorporated in the full model.

Model 7 combined ADown and SEAMHead, achieving the largest computational cost reduction among the two-module combinations, with parameter count and FLOPs reduced by 23.89% and 17.90%, respectively. Precision increased by 0.46 percentage points, recall increased by 0.42 percentage points, mAP_50_ increased by 0.14 percentage points, and mAP_50–95_ increased by 0.05 percentage points, all falling within the comparable range. These results demonstrate that the combination achieves substantial lightweight compression while maintaining detection accuracy at a level comparable to the baseline.

The complete three-module Model 8 achieved a precision of 95.92%, a recall of 95.66%, an mAP_50_ of 97.57%, and an mAP_50–95_ of 54.93%. Relative to the baseline YOLOv11, precision increased by 0.71 percentage points, classified as modest improvement under the unified interpretation framework. Recall increased by 0.13 percentage points, mAP_50_ increased by 0.45 percentage points, and mAP_50–95_ increased by 0.25 percentage points, all falling within the comparable range. The complete model operated at a computational cost close to the minimum among all ablation variants, with parameter count reduced to 20.70M and FLOPs to 71.4G, representing reductions of 18.13% and 17.55% relative to the baseline. While Model 7 with ADown and SEAMHead achieved marginally lower parameter count of 19.24M and FLOPs of 71.1G, the complete Model 8 provided a more favorable overall accuracy-efficiency balance with mAP_50_ of 97.57% compared with 97.26% for Model 7. Although this difference of 0.31 percentage points falls within the comparable range under the unified framework, the complete model offers a practical advantage given the near-identical computational cost and higher overall accuracy. These results confirm that the three-module combination preserves detection accuracy at a level comparable to the baseline while achieving near-minimal computational cost among all ablation variants, demonstrating that coordinated lightweight modifications across multiple network stages can jointly optimize the accuracy-efficiency trade-off.

Overall, the ablation results demonstrate that each proposed module makes a distinct contribution to model performance within the context of the coordinated pipeline. ADown establishes the lightweight foundation by reducing parameters by over 23% with accuracy differences falling entirely within the comparable range. AIFI strengthens global feature modeling, yielding a meaningful precision improvement of 1.39 percentage points with no measurable increase in inference latency. SEAMHead enhances target discrimination at the prediction stage, yielding a modest mAP_50_ improvement of 0.92 percentage points while contributing marginally to model compression. When deployed jointly, the three modules achieve the most favorable trade-off between detection accuracy and computational efficiency among all ablation variants, with all accuracy differences relative to the baseline falling within the comparable to modest improvement range while achieving near-minimal computational cost. The primary value of the coordinated design lies in demonstrating that substantial lightweighting can be achieved without meaningful accuracy degradation, which is a prerequisite for edge deployment on resource-constrained platforms. The 18.13% reduction in parameters and 17.55% reduction in FLOPs, achieved without meaningful accuracy loss across any of the four evaluated metrics, directly translate into the deployment advantages demonstrated in Section 5.5, including reduced engine file size, improved production-line throughput, and tighter scheduling margins on the NVIDIA Jetson Orin NX platform. **Figure 9** presents a qualitative comparison of detection results between the baseline YOLOv11 and the improved SAD-YOLOv11 under the same conditions.

**Figure 9:**
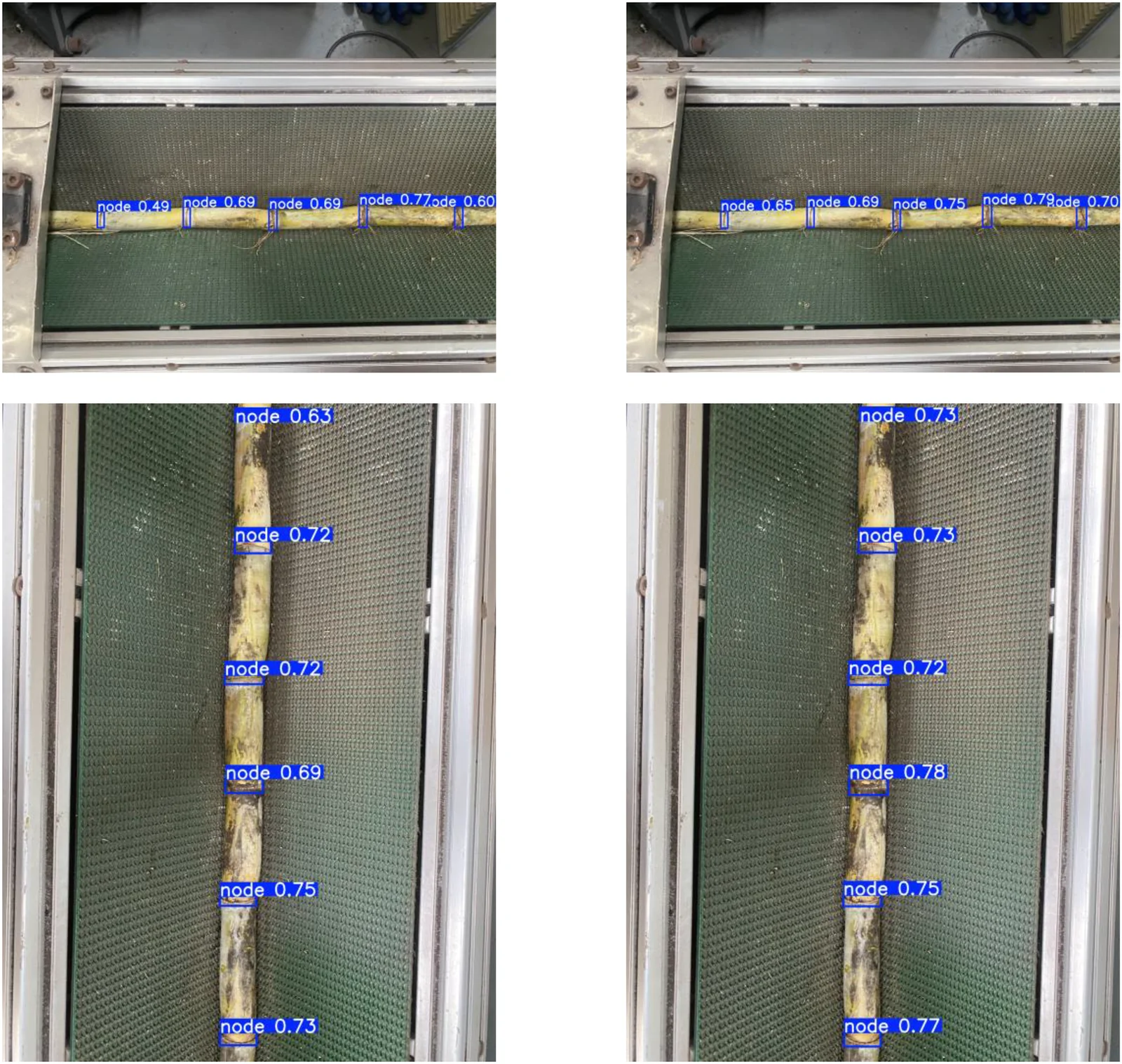
Comparison of detection results of different models under the same conditions. Left: baseline YOLOv11, Right: improved SAD-YOLOv11.

### 5.3 Comparison with mainstream detection models

To evaluate the overall performance of SAD-YOLOv11 more comprehensively, several mainstream object detection models were selected for comparison, including YOLOv5, YOLOv8, YOLOv10, YOLOv11, YOLOv12, YOLOv13, and YOLOv26. All models were trained and tested on the same dataset under the same experimental environment. The evaluation metrics included precision, recall, mAP50, mAP50-95, parameter count, floating-point operations, and FPS.

To ensure reproducibility and fairness, the specific configurations of all comparison models are detailed as follows. All models, including the baseline YOLOv11l and six comparison models, namely YOLOv5l, YOLOv8l, YOLOv10l, YOLOv12l, YOLOv13l, and YOLOv26l, were implemented through a locally customized Ultralytics framework based on version 8.4.24. The large-scale variants were used consistently across all comparisons. All models were trained from scratch without loading pretrained weights and under identical hyperparameter settings, including an input size of 640 by 640, a batch size of 64, a maximum of 10000 epochs, an early stopping patience of 50, a momentum of 0.937, a weight decay of 0.0005, and an initial learning rate of 0.01. The YOLOv12 architecture was defined by the model configuration provided in the Ultralytics framework, incorporating A2C2f attention modules for enhanced feature interaction. The YOLOv13 architecture was integrated into the same framework based on the design proposed by Lei et al., incorporating the HyperACE module for hypergraph-enhanced adaptive correlation and the FullPAD paradigm for full-process aggregation and distribution, with its custom modules implemented in the framework’s extra_modules component. The YOLOv26 model was trained and evaluated through the same unified pipeline using the standard Detect detection head with Distribution Focal Loss and conventional NMS post-processing, ensuring complete methodological consistency with all other compared models. It should be noted that the official YOLOv26 architecture introduces a dual detection head design for NMS-free end-to-end inference; however, this feature was not available in the framework version used in this study, and all models were therefore evaluated under the same conventional NMS-based inference pipeline.

The results are shown in **Table 2** and visualized in **Figure 10**, which provides an intuitive comparison of detection accuracy and computational efficiency across all eight models. **Figure 10** is plotted from the five-run mean across all independent training runs, including a scatter plot of mAP50 versus FLOPs, a grouped bar chart of accuracy metrics, a horizontal bar chart of parameter count, and a dumbbell chart comparing SAD-YOLOv11 against each baseline model across four accuracy metrics. Error bars indicate the standard deviation across the five runs. The complete five-run mean and standard deviation values are reported in **Table 2**.

**Figure 10:**
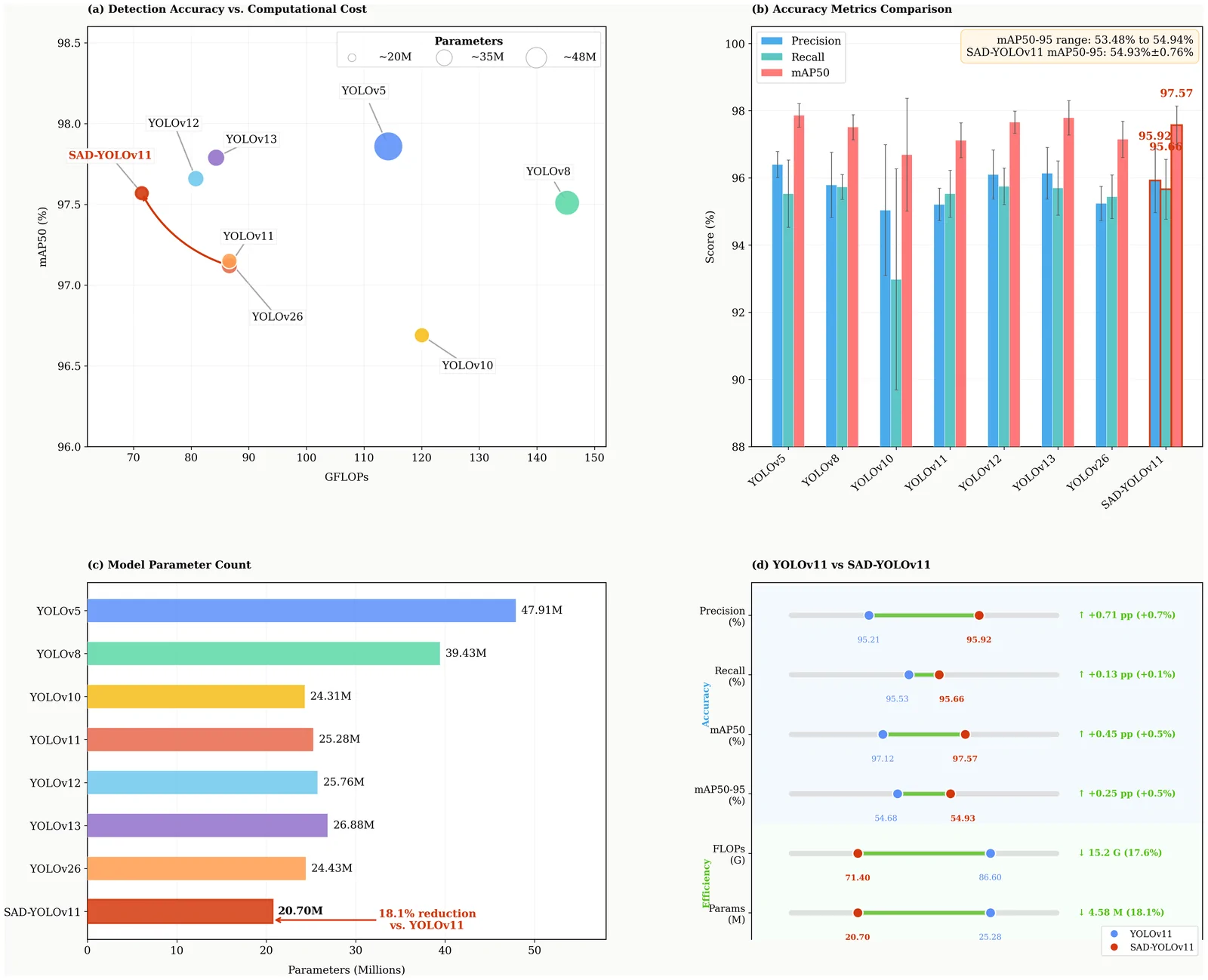
Visualization of the performance comparison between SAD-YOLOv11 and seven mainstream detection models. All numerical values are averaged over five independent training runs with different random seeds; error bars indicate standard deviation. **(a)** Scatter plot of mAP50 versus FLOPs, with bubble size encoding parameter count. **(b)** Grouped bar chart of Precision, Recall, and mAP50. **(c)** Horizontal bar chart of parameter count. **(d)** Dumbbell chart comparing SAD-YOLOv11 against each baseline model across four accuracy metrics, where each connected dot pair indicates the metric values of SAD-YOLOv11 and the corresponding comparison model, with the line segment directly conveying the direction and magnitude of the difference. All variants and metric values correspond directly to those reported in **Table 2**.

As shown in **Table 2**, SAD-YOLOv11 offers a favorable balance between detection accuracy and model lightweighting. In terms of accuracy, its precision of 95.92%, recall of 95.66%, and mAP_50_ of 97.57% are within the performance range of all compared mainstream models. Relative to the baseline YOLOv11, precision increased by 0.71 percentage points, classified as modest improvement, while recall increased by 0.13 percentage points and mAP_50_ increased by 0.45 percentage points, both classified as comparable performance under the unified interpretation framework. In mAP_50_, SAD-YOLOv11 is comparable to YOLOv8 at 97.51% with a difference of 0.06 percentage points, and comparable to YOLOv5 at 97.86% with a difference of 0.29 percentage points, both within the comparable range. The model exceeds YOLOv10 at 96.69% by 0.88 percentage points, classified as modest improvement. Differences relative to YOLOv12 at 97.66% and YOLOv13 at 97.79% are 0.09 and 0.22 percentage points respectively, both within the comparable range. In the more demanding mAP_50–95_ metric, SAD-YOLOv11 achieved 54.93%, which is comparable to the baseline YOLOv11 at 54.68% and within the range of all compared models spanning from 53.48% for YOLOv10 to 54.94% for YOLOv8, indicating that the model maintains detection performance across the full range of IoU thresholds at a level consistent with mainstream detection architectures.

The distinguishing advantage of SAD-YOLOv11 lies in its computational efficiency. In terms of lightweighting, SAD-YOLOv11 had 20.70M parameters and 71.4G FLOPs, representing reductions of 18.13% and 17.55% relative to the baseline YOLOv11l. Compared with YOLOv5 and YOLOv8, its parameter count was reduced by 56.81% and 47.52%, respectively. Its FLOPs of 71.4G were substantially lower than those of YOLOv5 at 114.2G, YOLOv8 at 145.2G, and YOLOv10 at 120.0G, and were also lower than YOLOv12 at 80.8G, YOLOv13 at 84.3G, and YOLOv26 at 86.6G. Among all compared models, SAD-YOLOv11 achieved the lowest parameter count and the lowest FLOPs while maintaining mAP_50_ within 0.5 percentage points of the best-performing comparison model YOLOv5, demonstrating that the coordinated lightweight modifications effectively reduce computational cost without meaningfully compromising detection accuracy. In terms of inference throughput on the RTX A6000 GPU, SAD-YOLOv11 achieved 145.46 FPS, which is comparable to YOLOv8 at 148.65 FPS and YOLOv10 at 142.80 FPS, and higher than YOLOv12 at 87.80 FPS and YOLOv13 at 67.52 FPS. It should be noted that the inference throughput of SAD-YOLOv11 on the workstation GPU is marginally lower than the baseline YOLOv11 at 154.19 FPS, which is attributable to the Transformer-based self-attention operations in the AIFI module that exhibit lower arithmetic intensity on GPU architectures compared with the purely convolutional SPPF module. However, this isolated throughput difference on the workstation did not translate into a deployment disadvantage on the edge platform, as demonstrated in the production-line simulation results in Section 5.5. Among all compared models, SAD-YOLOv11 maintained mAP_50_ at a competitive level within 0.5 percentage points of the best-performing model while achieving the lowest FLOPs and the lowest parameter count. Overall, SAD-YOLOv11 maintained detection accuracy at a level comparable to the highest-performing mainstream models while achieving the most substantial reductions in model size and computational cost, suggesting that its practical value lies primarily in the favorable accuracy-efficiency trade-off rather than in accuracy superiority alone. As shown in **Figure 11**, these results suggest clear practical value for sugarcane stem node detection on edge devices.

**Figure 11:**
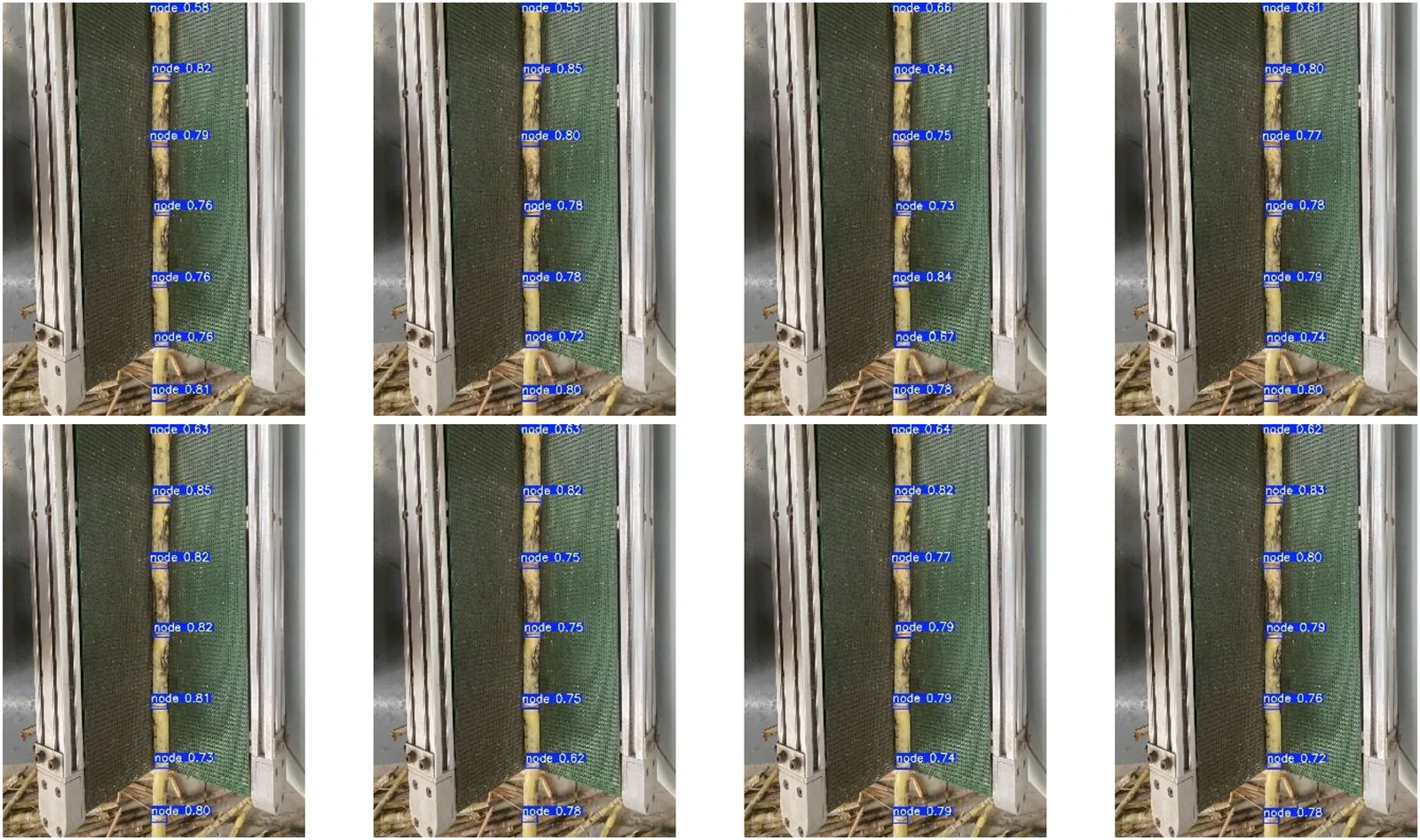
Detection results of mainstream detection models.

### 5.4 Robustness comparison under motion blur

To evaluate the detection robustness of the improved SAD-YOLOv11 model under degraded image quality, the images in the independent test set were quantitatively assessed for blur severity using the Laplacian variance metric, which computes the sum of squared second-order gradients of the image with lower values indicating stronger blur and reduced edge sharpness. To ensure that the robustness evaluation was free from training data contamination, only the 120 test images, which were held out during all model training, validation, and checkpoint selection procedures, were included in the detection evaluation. The 25th and 75th percentiles of the Laplacian variance distribution across the complete dataset of 1200 images, corresponding to values of 110.37 and 305.86 respectively, were used as classification thresholds to define three blur levels. This threshold calibration based on the full dataset ensured that the classification boundaries reflected the overall image quality distribution. Based on these thresholds, images with a Laplacian variance of 305.86 or above were classified as clear, images between 110.37 and 305.86 as mild motion blur, and images below 110.37 as severe motion blur.

The resulting test subsets consisted of 27 clear images containing 145 target instances, 60 mild blur images containing 317 target instances, and 33 severe blur images containing 167 target instances. The mean instances per image were 5.37, 5.28, and 5.06 for the clear, mild, and severe subsets respectively, confirming that the annotation density remained consistent across blur levels. **Table 3** summarizes the statistics of each blur subset.

**Table 3:** Statistics of the motion blur test subsets defined by Laplacian variance thresholds applied to the independent test set.

Subset	Images	Target instances	Instances per image	Laplacian variance mean	Laplacian variance std	Laplacian variance range
Clear	27	145	5.37	424.01	76.59	316.2 to 617.3
Mild motion blur	60	317	5.28	191.88	56.37	110.9 to 304.2
Severe motion blur	33	167	5.06	76.37	14.70	48.1 to 105.9

Motion blur in these images was introduced primarily through involuntary camera movement inherent to handheld acquisition with a mobile phone operated in automatic mode, in which parameters such as shutter speed and ISO are dynamically adjusted by the device software rather than manually controlled by the operator. As a result, the specific exposure settings for each image were not recorded, which limits the ability to characterize the blur in standard photographic parameters. Comparative experiments were conducted using YOLOv11 as the baseline model, employing precision, recall, mAP50, and mAP50–95 as the core evaluation metrics. The experimental data are presented in **Table 4**.

**Table 4:** Performance comparison of sugarcane node detection between YOLOv11 and SAD-YOLOv11 under varying degrees of motion blur.

Test scenario	Model	Precision/%	Recall/%	mAP_50_/%	mAP_50-95_/%
Clear	YOLOv11	97.42	98.00	98.51	60.79
Clear	SAD-YOLOv11	97.83	98.55	98.96	63.96
Mild motion blur	YOLOv11	97.33	95.92	97.76	56.56
Mild motion blur	SAD-YOLOv11	97.08	95.37	97.81	57.18
Severe motion blur	YOLOv11	93.06	92.79	94.65	54.52
Severe motion blur	SAD-YOLOv11	94.70	93.68	95.88	55.43

Results in this table were obtained by evaluating the best checkpoint from the training run with random seed 0 for each model on the same blur subsets. This single-model protocol provides a directional comparison of robustness under image degradation rather than statistically confirmed absolute differences.

As shown in **Table 4**, both models performed well under clear imaging conditions with comparable detection metrics. SAD-YOLOv11 achieved precision of 97.83% and recall of 98.55%, compared with 97.42% and 98.00% for the baseline, differences of 0.41 and 0.55 percentage points respectively. The mAP_50_ reached 98.96%, representing an increase of 0.45 percentage points over the baseline at 98.51%, which falls below the 0.5 percentage point threshold for comparable performance. The mAP_50–95_ reached 63.96% compared with 60.79% for the baseline, an increase of 3.17 percentage points exceeding the meaningful improvement threshold. These results suggest that under ideal imaging conditions, the two models exhibit comparable core detection performance, while SAD-YOLOv11 may provide marginally tighter bounding box alignment at higher IoU thresholds.

Under mild motion blur, both models experienced moderate declines in detection metrics. SAD-YOLOv11 achieved an mAP_50_ of 97.81% compared with 97.76% for the baseline, a difference of 0.05 percentage points that falls within the comparable range. Precision decreased by 0.25 percentage points and recall decreased by 0.55 percentage points relative to the baseline, both within the comparable range. The mAP_50–95_ reached 57.18% compared with 56.56% for the baseline, a difference of 0.62 percentage points classified as modest improvement. These results confirm that under mild blur conditions, the two models exhibit comparable core detection performance.

Under severe motion blur, images suffer a significant loss of high-frequency edge features, and the performance decline of both models becomes more pronounced. The baseline YOLOv11 achieved a precision of 93.06%, a recall of 92.79%, and an mAP_50_ of 94.65%. SAD-YOLOv11 achieved a precision of 94.70%, a recall of 93.68%, and an mAP_50_ of 95.88%. The precision improvement of 1.64 percentage points and the mAP_50_ improvement of 1.23 percentage points both exceed the 1.0 percentage point threshold for meaningful improvement under the unified interpretation framework, while the recall improvement of 0.89 percentage points falls within the modest improvement range. The mAP_50–95_ reached 55.43% compared with 54.52% for the baseline, a difference of 0.91 percentage points classified as modest improvement. Notably, severe blur is the only condition under which mAP_50_ differences exceed the meaningful improvement threshold. These results indicate that SAD-YOLOv11 maintains detection performance comparable to the baseline under clear and mild blur conditions, while demonstrating a meaningful improvement in mAP50 under severe blur. The lightweight architecture does not compromise robustness to image degradation and shows a modest advantage under severe blur conditions. It should be noted that the motion blur in this study originated from involuntary camera movement during handheld acquisition rather than from relative camera-object motion during conveyor belt transport. Therefore, the direct generalizability of these findings to industrial conveyor belt conditions with motion blur induced by relative movement at high transport speeds requires additional validation. Nevertheless, the results demonstrate that the model maintains favorable detection characteristics under the types of image degradation that are likely to arise in practical field deployment scenarios.

To further illustrate the detection behavior of the two models under degraded image conditions, several representative false positive and false negative cases are examined using **Figure 12** and **Table 5**. **Figure 12** presents the detection results of both models on the same set of blurred images. The left column shows the outputs of the YOLOv11 baseline model, and the right column shows the outputs of SAD-YOLOv11 on the corresponding blurred images. Blue boxes indicate sugarcane stem nodes, and the numbers beside the boxes are the corresponding confidence scores. **Table 5** provides a detailed case-by-case comparison.

**Figure 12:**
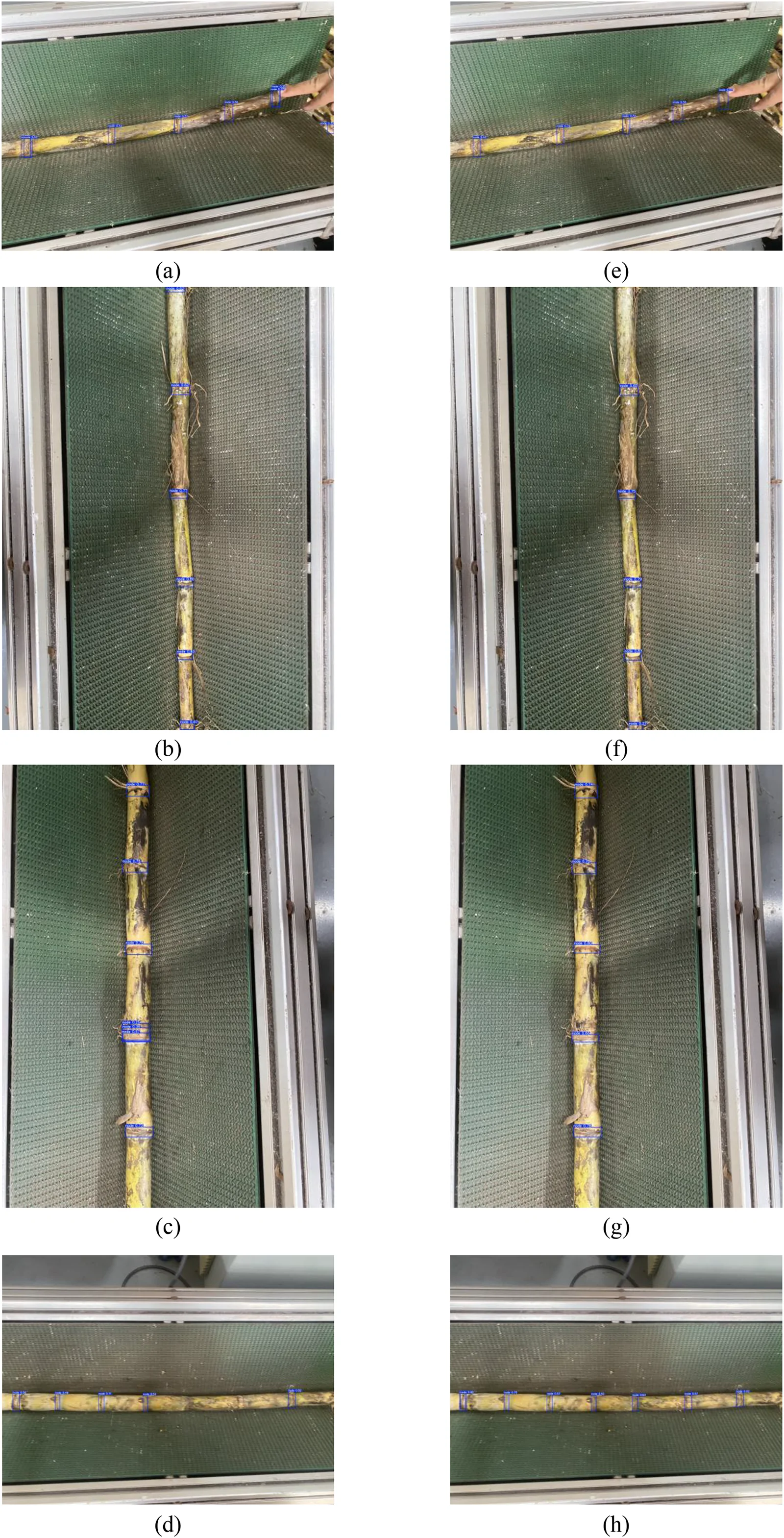
Comparison of sugarcane stem node detection results under motion blur. Panels **(a–f)** show three representative test cases under mild motion blur. Panels **(g, h)** show one representative test case under severe motion blur. In each pair, the left panel shows the output of the baseline YOLOv11 and the right panel shows the output of the improved SAD-YOLOv11. Blue boxes indicate sugarcane stem nodes and the numbers beside each box are the corresponding confidence scores. Panel labels a to h are shown in the figure and correspond to the error case analysis in **Table 5**.

**Table 5:** Detailed comparison of representative error cases under motion blur.

Panel	Blur level	GT	Baseline det	Baseline TP	Baseline FP	Baseline FN	SAD Det	SAD TP	SAD FP	SAD FN
(a),(e)	Mild	5	8	5	3	0	5	5	0	0
(b),(f)	Mild	4	6	4	2	0	5	4	1	0
(c),(g)	Mild	5	7	5	2	0	5	5	0	0
(d),(h)	Severe	7	5	5	0	2	8	7	1	0

TP, FP, and FN are determined by greedy IoU-based matching at a threshold of 0.50 between each model’s predicted bounding boxes and the ground truth annotations for the corresponding image. TP denotes true positives where a predicted box matches a ground truth box. FP denotes false positives where a predicted box does not match any ground truth box. FN denotes false negatives where a ground truth box is not matched by any predicted box. Det denotes the total number of detected bounding boxes. The cases were selected to illustrate representative detection behavior under different blur levels and are not random samples.

Under mild motion blur, both models were generally able to detect the stem nodes present in the images. In the selected illustrative cases, the baseline model produced more unmatched detections than SAD-YOLOv11. In panels a and e, the baseline produced 3 false positives against 5 ground truth targets with confidence scores ranging from 0.28 to 0.73, while SAD-YOLOv11 achieved 5 true positives with 0 false positives and confidence scores ranging from 0.42 to 0.73. In panels b and f, the baseline produced 2 false positives while SAD-YOLOv11 produced 1 false positive, with both models detecting the 4 ground truth targets. In panels c and g, the baseline produced 2 false positives while SAD-YOLOv11 achieved 5 true positives with 0 false positives. These observations suggest that under mild blur, the baseline model generates more redundant bounding boxes, though SAD-YOLOv11 also produced at least 1 false positive in one of the three cases. Under severe motion blur, the baseline model exhibited 2 missed detections in panels d and h, detecting 5 of 7 ground truth targets with confidence scores between 0.49 and 0.71. SAD-YOLOv11 detected 8 targets with confidence scores between 0.49 and 0.75, achieving 7 true positives with 1 false positive and no missed detections. While SAD-YOLOv11 produced 1 false positive in this case, it maintained higher detection completeness than the baseline under severe degradation.

These qualitative observations from the selected illustrative cases are consistent with the quantitative results in **Table 4**. Under clear and mild blur conditions, the differences between the two models fall within the comparable range for the primary mAP_50_ metric. In the selected mild blur cases, the baseline produced more unmatched detections than SAD-YOLOv11, although SAD-YOLOv11 also generated at least 1 false positive in one case. Under severe blur, SAD-YOLOv11 demonstrates a meaningful improvement in mAP_50_ of 1.23 percentage points. In the selected severe blur case, SAD-YOLOv11 achieved higher detection completeness with 7 of 7 ground truth targets detected compared with 5 of 7 for the baseline, while also producing 1 false positive. However, it is important to note that the motion blur observed in this study originated from handheld camera movement rather than from object motion on a moving conveyor belt. The generalizability of these robustness findings to industrial conveyor belt conditions with motion blur induced by relative camera-object movement at high transport speeds would require additional validation. Furthermore, the image acquisition did not record camera exposure settings, which limits the ability to characterize the blur conditions in terms of standard photographic parameters.

### 5.5 Edge deployment and TensorRT-FP16 quantization experiment

The preceding training, validation, and testing experiments were all conducted on a high-performance workstation equipped with an Intel Xeon Gold 6226R processor and an NVIDIA RTX A6000 GPU. While this hardware configuration ensured sufficient computational resources for model optimization and fair comparison among detection architectures, it is not representative of the resource-constrained environments encountered in practical agricultural deployment. As discussed in the Introduction, existing detectors with large parameter counts and substantial computational demands constitute a primary bottleneck for their deployment on edge devices, where computing power is typically limited to 0.5–100 TOPS and power budgets are tightly constrained. Complex network architectures that depend heavily on high-performance GPU hardware fail to meet the low cost and high efficiency requirements of agricultural production. The lightweight improvements in SAD-YOLOv11 include reduced parameter count, lower FLOPs, and streamlined module design. Whether these improvements translate into tangible deployment benefits under resource-limited conditions cannot be determined solely from workstation-based experiments. It is therefore necessary to evaluate the model on a representative embedded edge platform and quantify its actual performance in terms of inference speed, latency, memory consumption, and other deployment-critical indicators. The following experiments were conducted entirely on an NVIDIA Jetson Orin NX edge device (Bao et al., 2026), which represents a typical hardware platform for intelligent agricultural machinery, thereby providing direct evidence for or against the practical deployability of SAD-YOLOv11 in real-world sugarcane cutting scenarios.

#### 5.5.1 Edge deployment platform

Edge deployment experiments were conducted on the NVIDIA Jetson Orin NX Developer Kit platform, equipped with an ARM processor (8-core, up to 2.0 GHz), an NVIDIA Ampere-architecture GPU (GA10B, 1024 CUDA Cores, up to 918 MHz), 16.0 GB LPDDR5 system memory (EMC frequency 3199 MHz), delivering 100 TOPS of AI computing performance. The software environment was built upon Ubuntu 20.04.6 LTS (kernel 5.10.104-tegra) with JetPack 5.1.1 (L4T 35.3.1), CUDA 11.4.315, cuDNN 8.6.0.166-1+cuda11.4, and TensorRT 8.5.2.2-1+cuda11.4. The input resolution was uniformly set to 640×640 pixels with a batch size of 1, consistent with the training configuration. All deployment tests were performed under the MAXN power mode to fully utilize the available hardware resources.

#### 5.5.2 Quantization comparison under FP32 and FP16

To evaluate the impact of TensorRT-FP16 quantization on both the detection accuracy and inference speed of SAD-YOLOv11, TensorRT inference engines were constructed under both FP32 and FP16 precision modes on the Jetson Orin NX platform. FP32 served as the full precision baseline, while FP16 was the adopted deployment scheme. Detection accuracy was validated on the 120 images test set, and inference latency was measured using the TensorRT trtexec profiling tool, which reports the mean GPU compute latency over multiple runs, excluding data preprocessing and postprocessing overhead. The detection accuracy and GPU compute latency under the two precision modes are summarized in **Table 6**.

**Table 6:** Comparison of detection accuracy, GPU compute latency, and engine size between SAD-YOLOv11 and the baseline YOLOv11 under different quantization settings on the Jetson Orin NX platform.

Model	Quantization	Precision /%	Recall /%	mAP_50_ /%	GPU latency /ms	Engine size /MB
YOLOv11	FP16	95.70	96.20	97.20	23.03	52.0
SAD-YOLOv11	FP16	97.09	96.82	97.85	26.06	44.7
SAD-YOLOv11	FP32	97.59	96.66	97.81	57.18	83.8

The TensorRT accuracy values were measured using a single trained model under deterministic inference, where identical weights and inputs yield identical outputs. This differs from the five-run evaluation protocol in **Table 1**, **2**. The SAD-YOLOv11 FP32 mAP_50_ of 97.81% differs from the five-run mean of 97.57% in **Table 2**, primarily due to differences in image preprocessing and NMS implementation between the deployment and validation pipelines. All FP32 versus FP16 comparisons for SAD-YOLOv11 use the identical deployment framework.

As shown in **Table 6**, FP16 quantization resulted in only marginal changes in detection accuracy compared with the FP32 baseline. The precision declined by 0.50 percentage points, from 97.59% to 97.09%, while the recall rose by 0.16 percentage points, from 96.66% to 96.82%. The mAP_50_ remained essentially unchanged, shifting from 97.81% to 97.85%, a difference of merely 0.04 percentage points. These minor differences fall within the range of normal experimental variation. While the precision decreased marginally by 0.50 percentage points, the recall slightly improved by 0.16 percentage points, and the mAP_50_ remained essentially unchanged with a difference of merely 0.04 percentage points. Taken together, these results confirm that FP16 half-precision quantization preserves the detection accuracy of SAD-YOLOv11 at a level practically indistinguishable from full precision inference.

In terms of inference efficiency, the FP16 engine achieved a GPU compute latency of 26.06 ms, which represents a 54.4% reduction compared with the FP32 baseline of 57.18 ms, corresponding to an approximately 2.19-fold acceleration of the GPU inference stage. Concurrently, the engine file size was compressed from 83.8 MB to 44.7 MB, representing a 46.7% decline in storage and memory footprint. These results demonstrate that FP16 quantization offers a favorable trade-off between precision preservation and computational efficiency. FP16 was therefore selected as the deployment precision for the model on the Jetson Orin NX edge device.

To further evaluate the deployment advantage of SAD-YOLOv11, the baseline YOLOv11 was also deployed on the same Jetson Orin NX platform under the identical TensorRT FP16 configuration. As shown in **Table 6**, SAD-YOLOv11 achieved a precision of 97.09%, a recall of 96.82%, and an mAP_50_ of 97.85%, exceeding the baseline by 1.39, 0.62, and 0.65 percentage points, respectively. The engine file size was reduced from 52.0 MB for the baseline to 44.7 MB for SAD-YOLOv11, corresponding to a 14.0% reduction in storage footprint. Although the baseline exhibited a lower isolated GPU compute latency of 23.03 ms compared with 26.06 ms for SAD-YOLOv11, this did not translate into higher end-to-end throughput, as further demonstrated in the production-line simulation results presented below.

#### 5.5.3 End-to-end inference latency analysis

To clarify the full pipeline inference performance improvement brought by FP16 quantization, this study conducted end-to-end latency tests for both FP32 and FP16 TensorRT engines on the Jetson Orin NX platform. The tests covered all 120 images in the test set, using CUDA Event for precise timing to separately quantify the time consumption of three stages: preprocessing, GPU computation, and postprocessing. The results are shown in **Table 7**.

**Table 7:** End-to-end inference latency breakdown for FP32 and FP16 engines on Jetson Orin NX.

Stage	FP32 latency /ms	Proportion	FP16 latency/ms	Proportion
Preprocessing (image resizing, normalization)	3.55 ± 1.13	4.9%	3.26 ± 0.30	8.1%
GPU computation (TensorRT inference)	53.21 ± 8.35	74.0%	22.50 ± 0.88	55.7%
Postprocessing (NMS, coordinate mapping)	15.18 ± 5.44	21.1%	14.65 ± 4.79	36.3%
End-to-end total	71.94 ± 11.20	100%	40.42 ± 5.13	100%
Equivalent FPS	13.9	—	24.7	—

As shown in **Table 7**, FP16 quantization reduced the end-to-end inference latency from 71.94 ms to 40.42 ms, achieving a full pipeline acceleration factor of 1.78, with the equivalent frame rate rising from 13.9 FPS to 24.7 FPS. Among the individual stages, GPU computation latency decreased from 53.21 ms to 22.50 ms, a decline of 57.7%, constituting the primary contributor to the overall speedup. In the FP32 engine, GPU computation accounted for 74.0% of the total latency, representing an evident performance bottleneck; after FP16 optimization, this proportion was reduced to 55.7%, and the bottleneck effect was substantially alleviated. The postprocessing stage, which was implemented in Python-based NMS, consumed a comparable duration under both precision modes at approximately 15 ms, with its proportion rising from 21.1% to 36.3%, thereby becoming the new bottleneck for the FP16 engine. This result suggests that further optimization of the NMS algorithm in a C++ deployment environment could lead to additional improvements in end-to-end frame rate.

#### 5.5.4 Production line real-time detection simulation

To evaluate the actual deployment performance of SAD-YOLOv11 under intelligent seed cane cutting equipment operating conditions, this study simulated three typical input frame rates, namely 30 FPS for slow speed, 45 FPS for medium speed, and 60 FPS for high speed, and conducted 60 second continuous frame inference tests using both FP32 and FP16 engines, recording actual throughput, frame drop rate, and latency distribution. These input frame rates represent the target detection cadence required by the cutting equipment rather than the physical transport speed of the conveyor belt, as the sugarcane stalks were stationary during image acquisition in this study. For clarity, it should be noted that the 24.7 FPS value reported in the preceding end-to-end latency analysis was calculated from single-frame inference latency measured within a Python-based pipeline, whereas the 37.1 FPS throughput in the production-line simulation was obtained from continuous streaming inference using a C++ runtime with an optimized NMS implementation. The results are shown in **Table 8**.

**Table 8:** Production-line real-time detection performance of SAD-YOLOv11 and the baseline YOLOv11 under varying input frame rates on the Jetson Orin NX platform.

Engine	Input frame rate/FPS	Actual FPS	Frame drop rate/%	Mean latency/ms	P95/ms	P99/ms
SAD-YOLOv11 FP32	30	19.6	50.0	51.07	52.44	53.74
SAD-YOLOv11 FP32	45	19.5	100.0	51.36	52.52	53.90
SAD-YOLOv11 FP32	60	19.4	100.0	51.56	52.77	55.27
SAD-YOLOv11 FP16	30	37.1	0.0	26.97	28.08	29.14
SAD-YOLOv11 FP16	45	37.2	19.6	26.84	27.43	28.30
SAD-YOLOv11 FP16	60	37.2	50.0	26.87	27.50	28.40
YOLOv11 FP16	30	33.6	0.0	29.75	32.09	33.72
YOLOv11 FP16	45	33.1	30.3	30.15	32.58	33.94
YOLOv11 FP16	60	33.0	50.7	30.30	32.73	33.89

Mean latency is the average end-to-end latency across all frames in 60 second continuous inference. P95 and P99 denote the latency thresholds below which 95% and 99% of frames fall, respectively; values closer to the mean indicate tighter latency distribution and more deterministic scheduling. Frame drop rate is the percentage of frames that are discarded because the end-to-end pipeline cannot complete processing before the next input frame arrives, measured over the 60-second continuous inference period. All experiments were conducted under the identical software environment, inference pipeline, and hardware configuration. The SAD-YOLOv11 FP32 and FP16 results demonstrate the effect of quantization optimization, while the YOLOv11 FP16 results enable a direct comparison between the proposed model and the baseline under the same deployment configuration.

The experimental results indicate that the FP32 engine failed to meet the real-time requirements at all tested input frame rates. Its actual throughput was only 19.4 to 19.6 FPS, and the frame drop rate already reached 50% under the 30 FPS slow input frame rate. Under the 45 FPS and 60 FPS input frame rates, the frame drop rate reached 100%, indicating a substantial loss of detection capability. The mean latency of the FP32 engine remained in the range of 51.07 to 51.56 ms across the three input frame rates, though its maximum observed latency reached 69.38 ms, reflecting considerable variance in individual frame latency.

In contrast, the FP16 engine demonstrated substantially improved real-time performance. Its actual throughput remained stable at 37.1 to 37.2 FPS across all tested speeds. Under the 30 FPS slow input frame rate, the frame drop rate was 0.0%, indicating that all frames completed inference within the 33.3 ms frame interval required by this input frame rate. The mean latency was 26.97 ms and the P99 latency was 29.14 ms, which falls below the 33.3 ms threshold with sufficient margin. Under the 45 FPS medium input frame rate, the frame drop rate was 19.6%, still offering reasonable detection coverage. Under the 60 FPS high input frame rate, the frame drop rate reached 50%, yet the actual throughput of 37 FPS was sufficient to cover the detection of a large majority of seed cane specimens.

Beyond throughput, the FP16 engine also exhibited high latency stability. Under all tested input frame rates, the mean inference latency remained within the range of 26.84–26.97 ms, with P95 not exceeding 28.08 ms, P99 not exceeding 29.14 ms, and the maximum observed latency at 36.38 ms. By comparison, the FP32 engine exhibited considerably greater latency fluctuations with a maximum of 69.38 ms. This indicates that FP16 quantization not only improved average speed but also enhanced the consistency of inference latency, which is of considerable importance for deterministic scheduling of production line cadence. The production-line simulation was also conducted for the baseline YOLOv11 under the same FP16 configuration. As shown in **Table 8**, at the 30 FPS input frame rate, both models maintained a zero frame drop rate. However, SAD-YOLOv11 achieved a throughput of 37.1 FPS compared with 33.6 FPS for the baseline, representing a 10.4% advantage. The mean latency of the baseline at 29.75 ms remained below the 33.3 ms frame interval, which is consistent with the observed zero frame drop rate under the buffering mechanism of the streaming pipeline. However, the P99 latency of the baseline was 33.72 ms, which indicates that approximately 1% of individual frames exceeded the 33.3 ms single-frame processing budget, suggesting that the baseline operates at the boundary of feasibility with minimal scheduling headroom. In contrast, SAD-YOLOv11 maintained a P99 latency of 29.14 ms, which falls below the 33.3 ms frame interval with approximately 4.2 ms of margin, providing more deterministic scheduling behavior. At the 45 FPS input frame rate, the baseline exhibited a frame drop rate of 30.3%, substantially higher than the 19.6% observed for SAD-YOLOv11. These results indicate that the lightweight architectural modifications in SAD-YOLOv11 provide tangible end-to-end deployment advantages over the original YOLOv11 baseline on the resource-constrained edge platform.

It is worth noting that the mean latency of 26.97 ms observed in the continuous streaming simulation is lower than the end-to-end latency of 40.42 ms reported in the preceding latency analysis. This discrepancy arises from differences in the software implementation of the two experiments. The end-to-end latency in **Table 7** was measured within a Python-based inference pipeline. In that pipeline, the postprocessing stage was dominated by NMS execution and accounted for approximately 14.65 ms. The continuous streaming simulation, however, employed a C++ runtime with an optimized NMS implementation, which substantially reduced postprocessing overhead. Both measurements are valid within their respective deployment contexts and complement each other by providing performance bounds across different software environments. From a practical standpoint, the continuous streaming results in **Table 8** more closely approximate the performance achievable in real embedded deployment scenarios, where compiled C++ runtimes are standard. The Python-based measurement in **Table 7** is nevertheless retained, as it provides a conservative upper bound estimate of latency and explicitly identifies the postprocessing stage as a candidate for further optimization in future work.

These results indicate that FP16 quantization is a necessary condition for real-time deployment of SAD-YOLOv11 on the tested edge platform. Under the input frame rates of 30 to 45 FPS tested in this study, the FP16 engine provided real-time detection capability, whereas the FP32 engine remained inadequate due to insufficient inference speed. Combined with the observation that FP16 quantization incurs negligible accuracy loss, FP16 was selected as the deployment precision scheme for SAD-YOLOv11.

#### 5.5.5 GPU memory consumption experiment

The Jetson Orin NX employs a unified memory architecture in which the CPU and GPU share 16.0 GB of LPDDR5 memory. The memory footprint of the model therefore directly determines the remaining available system memory and the capacity for concurrent tasks. In this study, the tegrastats tool with a 50 ms sampling interval was used to record the system memory usage of both FP32 and FP16 engines during continuous inference over 10 rounds, with 120 images per round. The results are shown in **Table 9**.

**Table 9:** Memory consumption comparison between FP32 and FP16 engines on Jetson Orin NX.

Quantization	Engine file size /MB	Pre-inference baseline RAM /MB	Peak inference RAM /MB	Inference memory increment /MB	Increment reduction vs. FP32
FP32	83.8	3816	9998	6182	—
FP16	44.7	4258	10007	5749	7.0%

RAM refers to the unified memory usage reported by tegrastats on the Orin NX, encompassing system processes, the Python runtime, the CUDA context, image buffers, and all memory overhead from the TensorRT engine. Inference memory increment is defined as peak inference RAM minus pre-inference baseline RAM, reflecting the memory growth attributable to the inference process itself.

As shown in **Table 9**, the FP16 engine file achieved a 46.7% compression, reducing from 83.8 MB to 44.7 MB, a decrease of 39.1 MB. This reduction lowers the model storage footprint, which is relevant for the storage constraints of edge devices, and may also reduce the I/O overhead during engine loading.

Regarding runtime memory, the inference memory increment of the FP16 engine was 5749 MB, a decrease of 433 MB corresponding to a 7.0% decline compared with the 6182 MB of the FP32 engine. This increment is primarily composed of TensorRT workspace allocation, intermediate activation tensors, and weight loading during inference. FP16 quantization compresses weight storage from 32-bit to 16-bit precision, thereby reducing the memory overhead of weight loading. However, due to internal TensorRT optimization strategies such as layer fusion and memory reuse, the memory difference between the two precision modes does not follow a strictly linear proportional relationship.

Both configurations satisfied the system memory constraint. Within the 16.0 GB unified memory of the Orin NX, the peak RAM during inference under both FP32 and FP16 engines was approximately 10 GB, leaving approximately 6 GB available for concurrent tasks including the upper-level control system, communication modules, and the operating system. The FP16 engine, with its additional 433 MB of memory savings over FP32, offers a greater margin for concurrent scheduling in deployment scenarios requiring simultaneous execution of other GPU-intensive tasks. Furthermore, a comparison with the baseline YOLOv11 under the same FP16 configuration showed that the YOLOv11 FP16 engine file measured 52.0 MB compared with 44.7 MB for SAD-YOLOv11, corresponding to a 14.0% reduction in storage footprint achieved by the proposed model. Both models exhibited comparable peak memory consumption during inference on the Orin NX, confirming that SAD-YOLOv11 satisfies the memory constraints of the target edge platform while offering a moderately smaller storage footprint than the baseline.

## 6 Discussion

The primary practical contribution of SAD-YOLOv11 is not accuracy improvement over existing models but rather the demonstration that coordinated lightweight modifications can reduce model complexity sufficiently to enable real-time edge deployment while preserving competitive detection accuracy. This contribution directly addresses the central challenge identified in the Introduction, namely that existing detectors optimized for high precision are characterized by large parameter counts and computational costs that preclude deployment on resource-constrained edge platforms. The experimental results support this contribution from three perspectives. First, the model maintains detection accuracy at a level comparable to the baseline YOLOv11 and other mainstream models while achieving the lowest parameter count and FLOPs among all compared architectures. Second, the model exhibits improved robustness under severe motion blur conditions where mAP_50_ improvement of 1.23 percentage points exceeds the meaningful improvement threshold. Third, physical edge deployment on the NVIDIA Jetson Orin NX platform achieves real-time throughput under tested input frame rates with zero frame drop rate at 30 FPS input frame rate.

The experimental results presented above indicate that SAD-YOLOv11 achieves a favorable trade-off between detection accuracy and computational efficiency for sugarcane stem node detection. The ablation study suggests that the overall performance of the coordinated design arises from the complementary functional roles of the three modules across different network stages. The ADown module provides the lightweight foundation by reducing parameters while preserving spatial detail. The AIFI module was positioned at the deepest backbone layer to model global contextual dependencies through self-attention. The SEAMHead module applies channel-spatial joint attention at the prediction stage to enhance target-relevant features. Across all ablation variants, the accuracy differences relative to the baseline fell within the comparable to modest improvement range, while the complete three-module model achieved near-minimal parameter count and FLOPs among the ablation variants. These results are consistent with the hypothesis that the three modules function as a lightweight feature extraction, semantic enhancement, and feature discrimination pipeline, although the individual contribution of each stage cannot be isolated from the performance metrics alone. It is also worth noting that while SAD-YOLOv11 achieved FLOPs of 71.4G, which is among the lowest across all ablation variants and only marginally above the minimum of 71.1G observed in Model 7, its GPU inference throughput of 145.46 FPS on the RTX A6000 was marginally lower than the baseline YOLOv11 at 154.19 FPS. This observation highlights that FLOPs alone do not determine actual inference speed, as different operators have different hardware execution efficiencies. The AIFI module introduces Transformer-based self-attention operations involving matrix multiplication and softmax computations, which may have lower arithmetic intensity and less favorable memory access patterns on GPU architectures compared with the pure convolutional operations in the baseline SPPF module. This distinction is relevant to deployment optimization, where operator-level hardware efficiency should be considered alongside theoretical computational cost.

Compared with existing methods for sugarcane stem node detection, SAD-YOLOv11 addresses limitations in two respects that are particularly relevant to intelligent seed cane cutting equipment. First, recent deep learning approaches for sugarcane stem node detection have improved accuracy under various conditions (Zhou et al., 2020; Chen et al., 2021; Wang et al., 2022; Zhou et al., 2022; Wen et al., 2023; Xie et al., 2023; Zheng et al., 2024; Wen et al., 2025), yet their large parameter counts and computational demands limit deployment on edge platforms with constrained computing resources. SAD-YOLOv11 addresses this limitation by reducing parameters to 20.70M and FLOPs to 71.4G while improving detection accuracy relative to the baseline, as confirmed by the ablation and comparison experiments. Second, the performance of existing models can decline substantially under image degradation caused by motion blur, which may arise from various sources including handheld camera movement and relative camera-object motion during transport. The robustness experiments on camera-motion blur images classified using Laplacian variance indicate that SAD-YOLOv11 maintains detection performance comparable to the baseline under clear and mild blur conditions, with a mAP50 improvement observed under severe blur. A relevant comparison can be made with EdgeSugarcane, a recently proposed lightweight sugarcane node detection method that also targets edge deployment on the NVIDIA Jetson Orin NX platform. EdgeSugarcane is based on YOLOv8 with a Triplet Attention module and TensorRT-FP16 quantization, achieving a precision of 93.5%, a recall of 80.0%, and an mAP of 87.0% on a dataset of 600 indoor and outdoor images. Under the same edge platform, EdgeSugarcane reported a single-frame GPU inference time of 1.5 ms but a total end-to-end latency of 66.5 ms, with preprocessing accounting for 60.6 ms of the total. In comparison, SAD-YOLOv11 achieved a precision of 95.92%, a recall of 95.66%, and an mAP_50_ of 97.57% based on five training runs, with an end-to-end latency of 40.42 ms on the same Orin NX platform and a production-line throughput of 37.1 FPS under C++ runtime. While the two studies differ in dataset composition, base model architecture, and experimental conditions, and therefore a direct comparison should be interpreted with caution, the results illustrate that SAD-YOLOv11 achieves higher detection accuracy with a lower end-to-end latency on the same Orin NX platform. This comparison serves to contextualize the deployment performance of SAD-YOLOv11 within the existing literature rather than to claim direct superiority under matched conditions.

The edge deployment experiments on the NVIDIA Jetson Orin NX platform further validate that the structural lightweighting achieved by SAD-YOLOv11 translates into tangible deployment advantages. Rather than relying solely on theoretical metrics such as parameter count and FLOPs, this study evaluated execution indicators directly relevant to practical deployment, including GPU compute latency, engine size, and unified memory footprint. The TensorRT-FP16 quantization scheme compressed the model storage footprint by 46.7% and accelerated GPU inference by a factor of approximately 2.19, while detection accuracy changes remained within the range of normal experimental variation. The production line simulation further demonstrated that the SAD-YOLOv11 FP16 engine achieved stable throughput with the 99th percentile latency remaining under 30 ms, indicating reliable deterministic scheduling for the tested input frame rates. Additionally, the latency discrepancy observed between the Python and C++ runtimes illustrates that the choice of deployment software environment can substantially influence end-to-end performance. In this study, the optimized C++ implementation reduced the postprocessing overhead that dominated the Python-based measurements, suggesting that software-level optimization complements algorithmic acceleration in achieving favorable deployment outcomes.

The direct edge deployment comparison between SAD-YOLOv11 and the baseline YOLOv11 further supports the practical value of the proposed lightweight modifications. In the isolated trtexec GPU benchmark, the baseline achieved a lower compute latency of 23.03 ms compared with 26.06 ms for SAD-YOLOv11. This difference is attributable to the Transformer-based self-attention operations in the AIFI module, which exhibit lower per-operator arithmetic intensity on GPU architectures compared with the purely convolutional SPPF module in the baseline. However, this isolated latency advantage of the baseline did not translate into higher end-to-end throughput. In the continuous streaming simulation at the 30 FPS input frame rate using the C++ runtime, SAD-YOLOv11 achieved an actual throughput of 37.1 FPS compared with 33.6 FPS for the baseline, with both models maintaining zero frame drop rates. The 10.4% throughput advantage of SAD-YOLOv11 can be attributed to its lower overall pipeline overhead, arising from reduced parameter count and FLOPs that lead to more efficient memory access patterns and lower intermediate tensor transfer costs across the end-to-end inference pipeline. More critically, while both models achieved zero frame drop rates at 30 FPS, the P99 latency of the baseline was 33.72 ms, which indicates that approximately 1% of individual frames exceeded the 33.3 ms single-frame processing budget. The mean latency of 29.75 ms remaining below the frame interval is consistent with the observed zero frame drop rate, as the streaming pipeline buffering can absorb occasional slow frames when the mean latency is below the interval. However, the P99 exceeding the frame interval indicates that the baseline operates with negative scheduling headroom at the tail of the latency distribution. In contrast, SAD-YOLOv11 maintained a P99 of 29.14 ms, which falls below the 33.3 ms frame interval with approximately 4.2 ms of scheduling margin, providing more deterministic inference timing throughout the entire latency distribution. At the 45 FPS input frame rate, the baseline exhibited a frame drop rate of 30.3%, substantially higher than the 19.6% for SAD-YOLOv11. Additionally, the engine file of SAD-YOLOv11 was 14.0% smaller at 44.7 MB compared with 52.0 MB for the baseline. These results collectively indicate that the coordinated lightweight modifications in SAD-YOLOv11, while introducing Transformer operations with lower isolated operator efficiency, achieve superior end-to-end deployment performance through reduced overall computational and memory requirements.

Beyond the deployment efficiency gains, it is also necessary to address the observed performance gap between mAP_50_ and mAP_50–95_ reported in the experimental results. A gap exists between the mAP_50_ of 97.57% and the mAP_50–95_ of 54.93% for the proposed model. The baseline YOLOv11 exhibits a comparable gap, with mAP_50_ of 97.12% and mAP_50–95_ of 54.68%, and all mainstream models tested in this study show similar patterns, with mAP_50–95_ values ranging from 53.48% for YOLOv10 to 54.94% for YOLOv8. This observation suggests that the gap is primarily related to the inherent geometric characteristics of the targets, and the proposed architecture does not exhibit a gap that is disproportionately larger than those observed in other models. As reported in the dataset analysis, over 85 percent of the stem node bounding boxes occupy less than 5 percent of the image area, with aspect ratios concentrated between 0.3 and 0.5. For such small, elongated targets, the IoU between a predicted bounding box and the ground truth is sensitive to localization offsets in the width direction. A bounding box that captures the general node region can achieve a 50% IoU due to partial overlap along the long axis. However, reaching IoU thresholds of 75% or above requires substantially more precise alignment along both axes, which is geometrically challenging for targets whose width spans only a small number of pixels at the model input resolution of 640 by 640. This sensitivity to minor localization offsets is consistent with observations reported in other small object detection tasks. Notably, all mainstream models tested on this dataset exhibit a comparable gap, with mAP_50–95_ values ranging from 53.48 to 54.94%, while SAD-YOLOv11 achieved an mAP_50–95_ of 54.93%, which is comparable to the highest value observed across all models. The gap therefore reflects the task’s inherent geometric difficulty rather than a localization weakness of the proposed model. Furthermore, it is important to consider which metric is most operationally relevant for the intended application. In the intended application of intelligent seed cane cutting, the detection model provides the cutter with the presence and approximate position of each stem node to avoid damaging the cane bud. The physical cutting tolerance zone is related to the dimensions of the bud region along the stalk axis. Under the model input resolution of 640 by 640 pixels, a detection that captures the general node region and achieves an IoU in the range of approximately 55% to 65% typically provides positioning information that is sufficient to guide the cutter within the bud-safe zone. The mAP_50–95_ metric places greater weight on IoU thresholds of 75% and above, which demands boundary precision beyond what may be required by the physical cutting tolerance. From this perspective, mAP_50_ may provide a more operationally relevant assessment of localization adequacy for the intended cutting task, while mAP_50–95_ offers supplementary information about the fine-grained boundary precision of the model. However, validating this interpretation would require integrating the detection model with an actual cutting system and measuring cutting quality outcomes, which is beyond the scope of the current study.

The primary contribution of this study is the demonstration that coordinated architectural modifications across multiple network stages can reduce model complexity for sugarcane stem node detection while maintaining detection accuracy at a level comparable to substantially larger baseline and mainstream models. The ADown, AIFI, and SEAMHead modules collectively reduce parameters by 18.13% and FLOPs by 17.55%, with all accuracy differences relative to the baseline falling within the comparable to modest improvement range under the unified interpretation framework. The model additionally exhibits improved robustness under severe motion blur conditions, where the mAP_50_ improvement of 1.23 percentage points exceeds the meaningful improvement threshold. The practical deployability of the model was further validated through physical edge deployment experiments on the NVIDIA Jetson Orin NX platform, achieving a production-line throughput of 37.1 FPS with zero frame drop rate at 30 FPS input frame rate.

Several limitations of the current study should be noted. The dataset was constructed around a single sugarcane variety, and the image acquisition environment was relatively structured with uniform industrial lighting and a fixed conveyor belt configuration. The generalization ability of the model therefore remains to be validated across multiple sugarcane varieties, growth stages, and more complex unstructured field conditions. Additionally, although the model achieves substantial compression relative to the baseline, the parameter count of 20.70M and FLOPs of 71.4G suggest that further compression may be possible for deployment on lower-power edge devices.

Future work will focus on four directions. The first is expanding dataset diversity to encompass a broader range of sugarcane varieties, growth stages, and field conditions to improve model generalization. The second is exploring advanced model compression techniques, such as channel pruning and knowledge distillation (Shi et al., 2020), with the goal of further reducing computational overhead while preserving detection accuracy. The third is integrating the detection model with actual seed cane cutting equipment to validate whether the achieved positioning accuracy is sufficient for bud-safe cutting quality. The fourth is validating the model’s detection robustness under motion blur induced by relative camera-object motion during actual conveyor belt transport, which differs from the handheld camera blur evaluated in this study and may present distinct degradation characteristics at varying transport speeds. These efforts may support deployment on lower-power edge devices and extend the evaluation to a wider range of intelligent sugarcane cutting and precision planting scenarios.

## 7 Conclusions

To address the high model complexity, loss of small target features, sensitivity to motion blur, and difficulty of edge deployment in sugarcane stem node detection, this study developed SAD-YOLOv11, a lightweight and accurate object detector based on YOLOv11. The model integrates three existing modules, the ADown average pooling downsampling module, the AIFI attention-based intra-scale feature interaction module, and the SEAMHead spatial attention enhanced detection head, into a coordinated pipeline. These coordinated modifications reduce parameter count and floating-point operations while improving the detection accuracy of sugarcane stem nodes. The main conclusions are as follows:

1. The coordinated lightweight modifications reduced model parameters by 18.13% and FLOPs by 17.55% relative to the baseline YOLOv11, while maintaining detection accuracy at a comparable level. Based on five independent training runs with different random seeds, SAD-YOLOv11 achieved a precision of 95.92%, a recall of 95.66%, an mAP_50_ of 97.57%, and an mAP_50–95_ of 54.93%, with standard deviations reported in **Table 2**. Relative to the baseline, precision increased by 0.71 percentage points, recall increased by 0.13 percentage points, mAP_50_ increased by 0.45 percentage points, and mAP_50–95_ increased by 0.25 percentage points, all falling within the comparable to modest improvement range under the unified interpretation framework adopted in this study. Comparisons with seven mainstream detection models confirmed that SAD-YOLOv11 achieved detection accuracy comparable to the best-performing models while maintaining the lowest computational cost, indicating a favorable trade-off between precision and efficiency suitable for resource-constrained edge deployment.
2. Robustness experiments under physically captured motion blur conditions showed that SAD-YOLOv11 maintained detection performance comparable to the baseline under clear and mild blur conditions, while achieving a mAP50 of 95.88% compared with 94.65% for the baseline under severe motion blur. Validation of detection robustness under industrial conveyor belt conditions with transport-induced blur remains a direction for future work.
3. Edge deployment on the NVIDIA Jetson Orin NX platform with TensorRT-FP16 quantization achieved a throughput of 37.1 FPS with zero frame drop rate under the 30 FPS input frame rate, outperforming the baseline YOLOv11 which achieved 33.6 FPS under identical conditions. The engine size was reduced by 46.7% relative to the FP32 model and by 14.0% relative to the baseline YOLOv11 FP16 engine. These results confirm the practical viability of the model for real-time deployment under the tested conditions.
4. The design principles underlying SAD-YOLOv11, namely lightweight downsampling for spatial detail preservation, global attention for semantic enhancement, and channel-spatial calibration for feature discrimination, may transfer to other agricultural object detection tasks involving small targets on structured backgrounds, such as double-bud intelligent planting, crop organ detection on conveyor systems, and quality inspection in agricultural processing lines. Further investigation across different crop species and operating environments is needed to validate this potential.

## Data Availability

The raw data supporting the conclusions of this article will be made available by the authors, without undue reservation.
